# An Exploration of the Universe of Polyglutamine Structures

**DOI:** 10.1371/journal.pcbi.1004541

**Published:** 2015-10-23

**Authors:** Àngel Gómez-Sicilia, Mateusz Sikora, Marek Cieplak, Mariano Carrión-Vázquez

**Affiliations:** 1 Intituto Cajal/CSIC, Madrid, Spain; 2 Instituto Madrileño de Estudios Avanzados en Nanociencia (IMDEA-Nanociencia),Madrid, Spain; 3 Institute of Science and Technology Austria, Klosterneuburg, Austria; 4 Instytut Fizyki PAN, Warsaw, Poland; Fudan University, CHINA

## Abstract

Deposits of misfolded proteins in the human brain are associated with the development of many neurodegenerative diseases. Recent studies show that these proteins have common traits even at the monomer level. Among them, a polyglutamine region that is present in huntingtin is known to exhibit a correlation between the length of the chain and the severity as well as the earliness of the onset of Huntington disease. Here, we apply bias exchange molecular dynamics to generate structures of polyglutamine expansions of several lengths and characterize the resulting independent conformations. We compare the properties of these conformations to those of the standard proteins, as well as to other homopolymeric tracts. We find that, similar to the previously studied polyvaline chains, the set of possible transient folds is much broader than the set of known-to-date folds, although the conformations have different structures. We show that the mechanical stability is not related to any simple geometrical characteristics of the structures. We demonstrate that long polyglutamine expansions result in higher mechanical stability than the shorter ones. They also have a longer life span and are substantially more prone to form knotted structures. The knotted region has an average length of 35 residues, similar to the typical threshold for most polyglutamine-related diseases. Similarly, changes in shape and mechanical stability appear once the total length of the peptide exceeds this threshold of 35 glutamine residues. We suggest that knotted conformers may also harm the cellular machinery and thus lead to disease.

## Author Summary

Misfolding and aggregation of several proteins are known to be related to neurodegenerative diseases. Among them, polyglutamine expansions are known to be responsible for at least 9 diseases, including Huntington. Nonetheless, the structural properties of these intrinsically disordered proteins are difficult to study using classical techniques because of their rapid fluctuations that result in high conformational polymorphism. Here, we use molecular dynamics simulations to study polyglutamines of different chain lengths, starting with short non-pathogenic ones, and study the independent structures they are able to form. We characterize all structures by their geometrical properties, connectivity, putative mechanical stability and residence time (life span). Similar to the findings of a previous study with polyvalines, only some of the conformers are similar to those found in natural globular proteins. Moreover, we find structures that contain knots in both polyglutamine and polyvaline 60-mers, although the former contains many more knotted conformers than the latter. We suggest that these knotted conformers may impair the cell machinery for degradation and eventually lead to toxicity.

## Introduction

Less than two thousands protein folds have been identified in nature [1, 2], indicating that similar folds can be adopted by large numbers of sequences. These folds have been characterized and classified in the CATH database [3]. Recently, Cossio *et al*. [4] considered a single sequence—polyvaline (polyV) 60-mer (denoted here as V_60_)—and generated, through all-atom simulations, an exhaustive database with 30 063 conformations. Interestingly, only a small fraction of the V_60_ conformations turned out to be CATH-like in that they had at least one similar structure in the CATH database. The similarity was assessed by a TM-score being higher than 45%. The score is obtained through an algorithm for protein comparison based on secondary structure alignment [5]. Thus they explored, in their own words [4], the universe of protein structures beyond the Protein Data Bank. They argued that there must be an evolutionary principle that favors shorter loops and directs the evolution to a certain spot in the universe of possible conformations.

Long polyV chains do not exist in nature. However, many proteins in eukaryotic cells contain homopolymeric tracts, defined as repetitions of the same residue. In particular, upon inspection of the revised human proteome stored in UniProt Knowledge Database [6], we found that 18.9% of the human proteome involves homopolymeric tracts of size 5 or greater, while the probability of one happening by chance is 6 ⋅ 10^−6^. Among these, the longest chains have been found for polyserine (polyS, 58 repeats, in the TNRC18 protein, with a random probability of 4 ⋅ 10^−76^) and polyglutamine (polyQ, 40 repeats, in FOXP2 protein, random probability of 9 ⋅ 10^−53^).

PolyQ chains are known to be responsible for several brain disorders, including Huntington disease (HD). HD is caused by a protein in the human brain known as huntingtin (HTT)—of, as yet, not fully elucidated function. HTT is known to be highly involved in development [7], and is thought to be related to gene expression regulation [8] and to anchoring or transport of vesicles [9]. A HTT mutant with an expansion of polyQ that exceeds the threshold of about 35-mer was linked to the disease [10]. Even though polyQ tracts have been extensively studied [11–13], the molecular physiopathology behind the connection between sequence length and disease remains elusive.

Another example of disease-related homopolymeric tracts is polyalanine (polyA), occurring in transcription factors. Expansions of the polyA tracts beyond certain thresholds (*e.g*. 19) have been recognized as the cause of congenital malformation syndromes, skeletal dysplasia and nervous system anomalies [14, 15]. The strong evolutionary conservation of the polyA tracts suggests the existence of critical structural or functional constraints [14]. It should be noted that in human proteins the polyA tracts are short compared to those of polyQ [15].

Here, we focus on polyQ chains of various lengths, Q_*n*_, where *n* goes from 16 to 80. The case of *n* = 62 was the subject of a recent single-molecule force spectroscopy study [16] that revealed a large conformational polymorphism (monitored as a spectrum of different breaking points and characteristic force-peak heights, up to 800 pN). The questions we ask are as follows: 1) can we explain this conformational polymorphism? 2) can polyQ tracts generate non-CATH-like conformers? 3) what are the structural and mechanical properties of the polyQ structures? In order to answer them, we follow a bias exchange molecular dynamics approach (BEMD) [17] also used by Cossio *et al*. [4]—one of the meta dynamics approaches—and explore the structural and dynamical properties of Q_*n*_ with a particular focus on Q_20_ and Q_60_, representative examples below and above the HD’s pathological threshold.

We take two perspectives in our analysis: 1) making comparisons of Q_60_ to V_60_ and to the similar-sized proteins from the CATH database; 2) investigating the changes in the physical properties of the conformations corresponding to Q_*n*_ as one varies *n*. The dynamical properties can be conveniently captured by their mechanical stability, as characterized by the characteristic force, *F*_max_, needed to unravel a structure by pulling by its termini at a constant speed, *v*_*p*_. This part of our studies makes use of a structure-based coarse-grained model [18, 19] to access the regime of near-experimental speeds and to deal with the large statistics. It should be noted that typical fluctuation times of these structures are much smaller than those needed to fully unravel them [20]. Thus, the results on *F*_max_ are merely indicators of the putative mechanical stability of each specific conformer that do not take the intrinsic evolution of the disordered protein into account.

We find that relatively large mechanical stability may arise not only from structures with large secondary structure content (SS, measured as the percentages of residues belonging to *α*-helices, *β*-strands and hydrogen-bonded turns) but also from those with SS of about 30%. Interestingly, we also find spontaneous generation of knotted structures for *n* = 60, which tend to be of a size of 36 residues, about HD’s threshold. This is a novel feature in neurotoxic proteins that needs further investigation.

## Methods

### Generation and selection of structures

Our BEMD [17] simulations were carried out using the GROMACS molecular dynamics package [21] and the PLUMED extension [22]. The force field used is AMBER99SB [23] and the implicit solvent model is the generalized Born surface area method [24]. The same force field has been used before in folding simulations with explicit solvent [17, 25], but implicit solvent is preferred in order to efficiently explore the energy landscape [4]. Structures were initialized randomly using the MODELLER software [26]: 10 off-template models were done for each protein; the models that contained knots were discarded and the remaining ones were minimized through up to 1000 steps of the steepest descent method followed by up to 4000 steps of the conjugate gradient algorithm [27]. The system which acquired the smallest potential energy after the two minimization stages was chosen for further studies.

In order to generate a variety of Q_*n*_ structures, we applied the BEMD method with six replicas, each with a different secondary structure bias: the first one with no bias; the next three with a preference to *α*-helix in the first, second and last third of the chain sequence; the fifth with a preference to anti-parallel *β*-strands and the last one with parallel *β*-strands. The method is explained in detail in the S1 Text.

We first obtained a number of conformations with a varying secondary structure content. To select the structures of interest, we followed the three-sieve protocol used in [4], described in the S1 Text, that yields structures with SS>30% which are temporally and structurally independent. From a 2 *μ*s simulation of Q_60_, 246 independent conformers were obtained from 953 time clusters. For Q_20_, a 0.66 *μ*s simulation resulted into 491 independent conformers out of 517 time clusters. Interestingly, half the simulation time for the short peptide yielded twice as many independent conformations as the longer one, which indicates higher polymorphism and faster dynamics in Q_20_. The procedure led to the emergence of some knotted structures even though there were no knots present in the initial homology-derived conformers.

After clustering, all independent structures underwent a minimization process of 10 000 steepest descent steps or until the maximum force between a pair of atoms was smaller than 0.25 J/(mol nm) so that the structure in the closest energy minimum is obtained. In this process, some of the residues may form or break contacts, thus changing their secondary structure content slightly. Therefore, even though the structures were selected with SS≥30% before the first clustering, some of the final structures may have a smaller SS content.

The structures of V_60_ were taken from ref. [4]. Their 50 *μ*s simulation yielded 30 063 time clusters. We have applied the structural clustering to this set and obtained 7076 independent conformers—as these were not available. All the independent structures generated are provided in the S1 Text.

Three previous works have explored the aggregation properties of Q_*n*_ by computer simulations. In the first of them, the focus is on the temporal stability of the structures and on the evaluation of their amyloidogenesis and fibrillation capabilites [28]. The second study explores the landscape of possible conformations by simplifying the structure of glutamine and generating a model that efficiently samples many conformations [29]. Finally, the third one applies replica exchange molecular dynamics to explore the dimerization of polyQ [30]. The three works show structures such as steric zippers and a *β*-helix, which have been found in our sampling among the strongest Q_60_ and Q_20_ conformations shown in S1 and S2 Figs. Furthermore, rod-like conformers with close to 100% *α*-helical content were also described in the aforementioned works and have likewise been found in this sampling. Our simulations did not find the mainly *β* conformations suggested in Ref. [30] because we consider monomers instead of dimers.

### Descriptors of the structures

Our structural analysis deals with several descriptors. One is the radius of gyration, *R*_*g*_, which characterizes the linear size of the molecule. Another is the *w* parameter which describes the shape [31, 32], which is defined through the diagonalization of the tensor of inertia and by making combinations of the three main radii such that a near-zero *w* corresponds to a globular shape, a positive *w* to an elongated one, and a negative *w* to a flattened object. The third descriptor is the SS parameter, which is determined by using the DSSP procedure [33]. This parameter is a sum of several ingredients: the *α*-helical content, the *β*-content (strands and bridges), and the hydrogen-bonded turn content.

The next two descriptors are *F*_max_ and ⟨*z*⟩—the average coordination number. The former relates to the dynamics directly, while the latter relates to it indirectly since *z* measures the number of residues a given residue interacts with. These interactions are of two kinds: through the peptide bond with the two nearest residues along the sequence and through contact interactions with residues which are not sequential neighbors. The contacts play a dynamical role in coarse-grained structure-based models but they can also be used as descriptors in all-atom models. The specific definition of the contacts we use is based on enlarged van der Waals spheres associated with the heavy atoms [18, 19] and the radii of the spheres are given in ref. [34]: a contact between two residues exists if there is at least one pair of heavy atoms with overlapping spheres.

In the structure-based model, we assign Lennard-Jones potentials of depth *ɛ* to these contacts (the potential minimum is at the distance between the C_α_ atoms in the reference structure) so that larger values of ⟨*z*⟩ are expected to correspond to more stable structures. Maxwell demonstrated [35] that large three-dimensional systems of particles with pairwise interactions are stable provided the ⟨*z*⟩ is bigger than 6. In particular, this finding has been shown to be consistent with the behavior of virus capsids [36]. In our case, the structures are much smaller than the capsids and, therefore, the threshold value of ⟨*z*⟩ is reduced, as explained in the S1 Text. Furthermore, the systems we study are also endowed with the local backbone stiffness—a four-particle interaction [37]—which favors the local chirality of the reference state. Thus non-zero values of *F*_max_ for structures with ⟨*z*⟩ smaller than 6 are allowed.

A stretching force (*F*) *vs*. displacement (*d*) curve may include articulated force peaks –which exceed the thermal noise level of about 0.1 *ɛ*/Å—before the *F* raises indefinitely due to stretching of the peptide bonds. The calculations are done at the temperature of 0.3 *ɛ*/*k*_B_, where *k*_B_ denotes the Boltzmann constant. The number of peaks is denoted by *n*_p_. *F*_max_ is defined as the height of the largest peak. If none exists then *F*_max_ is defined to be zero, even though it could take any value below the baseline of the curve (around 0.4 *ɛ*/Å). We simulate stretching at *v*_*p*_ of 5 ⋅ 10^−3^ Å/*τ*, where *τ* is of order 1 ns. Experimental *v*_*p*_’s are typically lower, *e.g*. 4 ⋅ 10^−6^ Å/ns in ref. [16]. We have calibrated [19] *ɛ* to be 110 pN Å (with a 25% error bar) by comparing theoretical and experimental values of *F*_max_ in 38 proteins (the theoretical results involved extrapolation to the experimental *v*_*p*_’s). The temperature in the coarse-grained simulations is in the vicinity of the room temperature.

Finally, one can consider the contact order (*CO*) as yet another structural descriptor. It is related to the number of contacts as well as the average distance along the sequence between the contacting residues, as defined in Ref. [38] as $CO=\frac{1}{L\cdot N}\sum_{k}S_{k}$, where *S*_*k*_ is the distance between the residues that form contact *k*, *L* is the number of residues in the protein and *N* the number of contacts. There is a question whether *CO* correlates with the folding time or not (see [39] and [40] for the arguments for and against it), so one may also inquire whether *F*_max_ correlates with *CO*. It seems unlikely that the free energy barrier to mechanical unfolding is of the same nature as the one for folding (or thermal unfolding) [41] but this does not preclude a correlation with *CO*. However, we do not find the correlation to be valid (see the S1 Text) which is consistent with the fact that the green fluorescent protein (PDB code 1GFL) has a bigger *F*_max_ than the I27 domain of titin (the PDB code 1TIT), 2.7 *vs*. 2.1 *ɛ*/Å [19], whereas its *CO* is smaller, 0.22 *vs*. 0.36.

## Results

### Properties of Q_60_ and Q_20_

We first consider the Q_60_ set, so that one can compare with V_60_ from [4] and with the experimental results on Q_62_ in [16]. S1 Fig shows structures corresponding to the top five values of *F*_max_. Similar figures for other sets studied are shown in S2 and S3 Figs. The values range between 2.1 and 2.3 *ɛ*/Å (approximately between 230 and 250 pN) which is of the order of what has been found—about 200 pN—for the I27 domain of titin [42, 43] at smaller *v*_*p*_’s. The fact that these values are much smaller than the ones found experimentally in [16] can be attributed to a small statistics, since the experimental systems yielded high force only with low probability (*p*(*F*_max_ > 200 pN) = 7 ± 6%). The figure also shows the corresponding *F* − *d* patterns together with distances at which particular contacts break down (the distance in the contact exceeds the reference distance by 50%) for the last time. The contacts are labeled by the sequential distances ∣*i* − *j*∣ between residues *i* and *j*. The number of force peaks varies between 1 and 4, corresponding to several substructures forming in each conformer. The third column of panels in S1 Fig provides the values of the relevant descriptors. *R*_*g*_ is seen to range between 11.05 and 14.20 Å and the values of *w* indicate that the fifth structure is elongated while the other four are nearly globular. The most stable structure of the five shown (the top row of panels) corresponds to the largest ⟨*z*⟩ (7.67), and SS (66.7%)—the secondary structure is, in this case, exclusively of the *β* type.

Interestingly, the second most robust structure, as judged by the value of *F*_max_, has very low ⟨*z*⟩ (5.33) and SS (28.3%).

**Fig 1:**
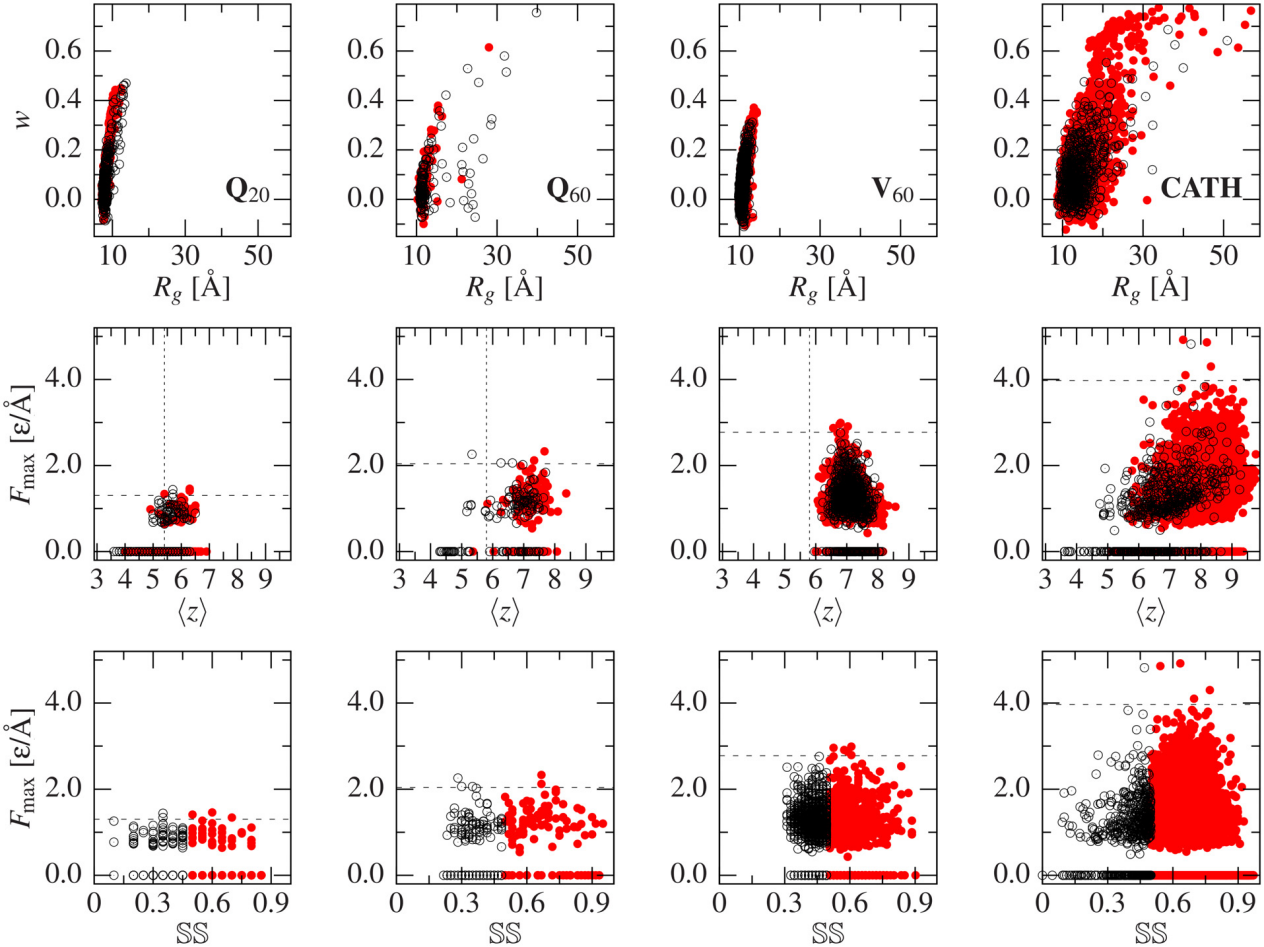
Scatter plot relating the specified variables for four differentsets, from left to right, Q_20_, Q_60_, V_60_ and CATH. The empty black points represent the conformers with less than 50% secondary structure content, while the filled red dots represent the more structured conformers. The vertical dotted lines in the middle panels mark the simply stiff limits of stability for each case (see the S1 Text). The conformers to the left of this line are more volatile. The horizontal dashed lines in the middle and bottom panels mark off the top five conformers with respect to the value of *F*_max_.

This system was compared to Q_20_, which is unrelated to disease and is close to Q_19_, which was studied experimentally [16]. The two left columns of Fig 1 refer to all structures in sets Q_20_ and Q_60_. In particular, the first row represents the geometries obtained on the *R*_*g*_—*w* plane. The convention we use is that we represent the data corresponding to structures with SS of at least 50% and those with lower SS by filled and open symbols, respectively. The structures from Q_60_ are seen to overlap with the region taken by Q_20_ but they also extend to much larger *R*_*g*_ and to bigger *w*. The largest value of *R*_*g*_ corresponds to a low SS, while high *w* can be achieved with any value of SS.

The two bottom rows of Fig 1 show scatter plots that compare the values of *F*_max_ in Q_60_ to those in Q_20_ when represented as a function of ⟨*z*⟩ and SS. S4 Fig provides a continuation in which *F*_max_ is plotted against the *α*-, *β*-, and turns (*τ*) content. It is clear that, for a given *n*, the mechanical stability is not related in any simple manner to either ⟨*z*⟩, *CO* or SS content. This is because typical high-force motifs include *β*-structured regions, while high ⟨*z*⟩ and SS can be achieved with *α*-structure and hydrogen-bonded turns [43]. S4 Fig shows that mechanical stability has no direct correlation to *α*-content or hydrogen-bonded turns (*τ*), and while most of the high *β*-content conformers lead to high forces, these can also be observed in cases with no *β*-content. Similar results are shown in the top panels of S5 Fig for the *CO*.

**Fig 2:**
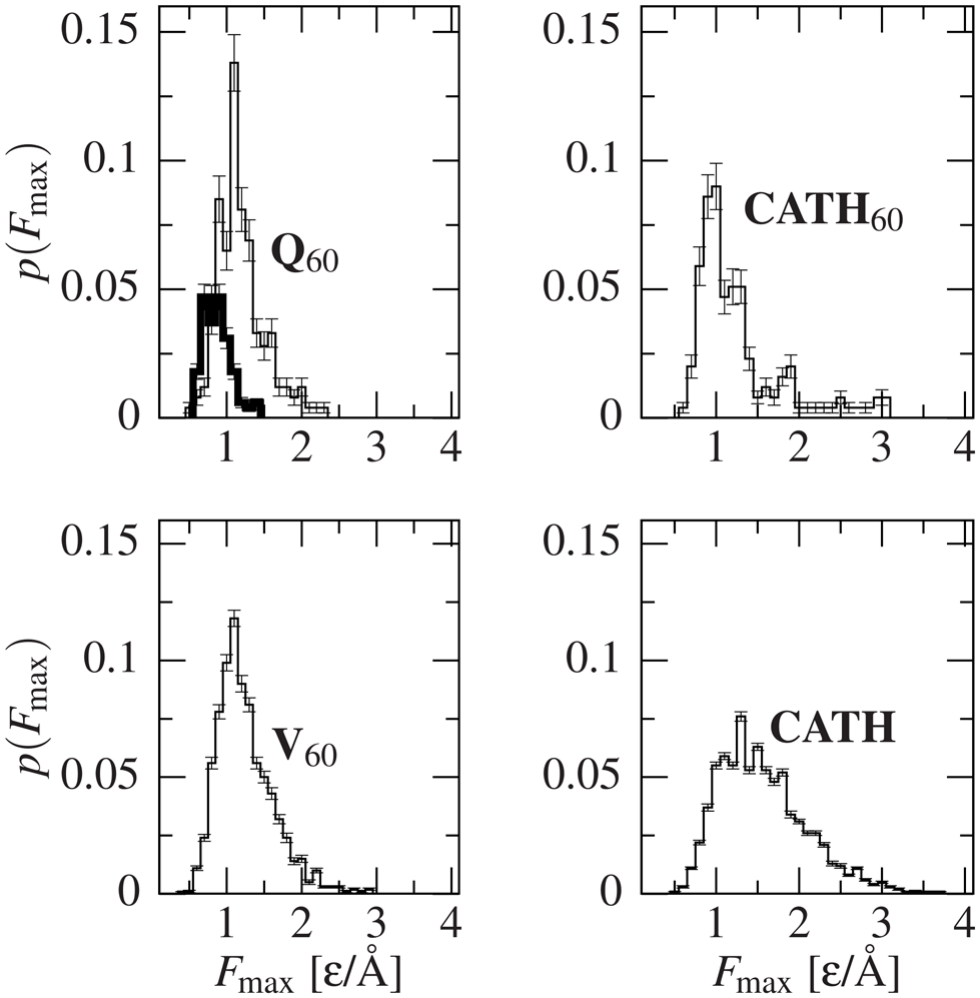
Distributions of *F*_max_ for the studied species. The top left panel shows the distribution for Q_20_ in a thick line. The conformations with no force peaks are not plotted in the histograms but contribute to normalization. The amount of such non-mechanostable conformers is (79 ± 2)% for Q_20_, (34 ± 3)% for Q_60_, (16.5 ± 0.2)% for V_60_, and (47 ± 3)% and (20.2 ± 0.5)% for CATH_60_ and CATH, respectively. The errors were computed using a bootstrapping method and the size of the error bar indicates the standard deviation.

Furthermore, in the top left panel of Fig 2 there is a comparison of the distributions of *F*_max_ for Q_60_ and Q_20_. We observe that although our BEMD simulations bias the chain towards the acquisition of SS, many conformers do not produce any articulated force peaks above the 10% noise level (*F*_max_ = 0). In particular, Q_20_ presents (79 ± 2)% of this kind of conformers, while Q_60_ only contains (34 ± 3)% of them. This result is consistent with the experimental data [16], where no force peaks were detected in Q_19_, while some were found in Q_62_. Remarkably, although the diversity in mechanical stability for Q_20_ is smaller than for Q_60_, the frequency of independent structure generation is greater in the former (see S6 Fig), so its conformational polymorphism should be higher. The volatility of each conformer, as assessed by ⟨*z*⟩ lower than their threshold, also agrees with this result, with (49 ± 2)% volatile conformers in Q_20_ *vs*. (13 ± 2)% in Q_60_.

Taken together, these results show that *F*_max_ is inherently different in Q_20_ and Q_60_ sets, even when SS is similar. This further points to *F*_max_ not being related to hydrogen-bonded turns, *α* helices and even *β*-strand content and SS, and also neither to *CO* nor to ⟨*z*⟩.

### Comparisons of Q_60_ to the remaining sets of structures

Fig 2 shows the normalized distributions of *F*_max_ within the Q_60_, Q_20_, V_60_, CATH_60_, and CATH sets. The distributions do not show the peak at *F*_max_ = 0, but its value is shown in the caption and contributes to the normalization. CATH_60_ is defined as structures containing between 57 and 63 residues and it contains 256 proteins. In order to exclude short peptides and most multidomain proteins we take CATH to represent all those proteins in the CATH database that are 40 to 250 residue long. This set comprises 5403 structures.

The characteristic forces grow on moving from Q_20_ to Q_60_. However, in all sets but Q_20_, the most probable *F*_max_ is about the same, 1.2 *ɛ*/Å, but the shapes of the distributions differ. The distributions are comparably broad for CATH and CATH_60_ and comparably narrower for Q_60_ and V_60_, indicating the role of the stronger compositional homogeneity in the latter two sets. The rougher look of the distribution for Q_60_ is likely due to the one order of magnitude smaller statistics. Furthermore, it should be noted that, among the systems of about 60 residues, CATH_60_ leads to the biggest number of situations with no force peaks, (47 ± 3)%; and V_60_ to the smallest, (16.5 ± 0.2)%.

Despite the similarity of the distribution of the forces between Q_60_ and V_60_, the geometrical character of structures in the two sets are distinct. Fig 1 shows that V_60_ conformers are more compact and less elongated than Q_60_ or CATH_60_. Furthermore, this figure indicates that size and shape of a chain need not be correlated.

Fig 1 further shows that most of the structures in the 60-sized sets, and also for CATH, come with ⟨*z*⟩ between 5.5 and 8.5. However, the largest values of *F*_max_ arise for ⟨*z*⟩ between 6.3 and 8.1 and many large ⟨*z*⟩ structures come with average or even small forces, including *F*_max_ of 0. Similarly, Fig 1 also demonstrates that large SS may come with low or zero forces and large *F*_max_ may arise when SS is at its lower range. Furthermore, the scattering of the data points in the *F*_max_–*CO* plane shown in Fig. [38] also points in the direction of statistical independence. This observation further proves that there is no correlation between *F*_max_ and ⟨*z*⟩, *CO* or SS. Interestingly, none of the independent V_60_ structures obtained from [4] have ⟨*z*⟩ below the volatility threshold, while (5.5 ± 1.4)% of the ones in CATH_60_ and (2.3 ± 0.2)% of CATH do. Our comparison reinforces the remarkable conclusion that *F*_max_ is unrelated to *CO*, SS or ⟨*z*⟩ and extends it to general globular proteins instead of being a property specific for polyQ. This is further discussed in the S1 Text, where the independence of *F*_max_ with the rest of parameters is proved.

### Life span of the structures

In order to test whether the coordination number is actually related to temporal stability, we performed 10 ns free-dynamics simulations on 100 structures chosen randomly from each set: Q_20_, Q_60_ and V_60_. We have studied the time dependence of RMSD relative to the initial structure and the last time that it fluctuated below 2 Å was recorded for each conformer as its time of residence (*t*_R_). Similarly, we define the escape probability (*P*_e_(*t*)) as the probability of leaving the initial conformation before time *t*.

**Fig 3:**
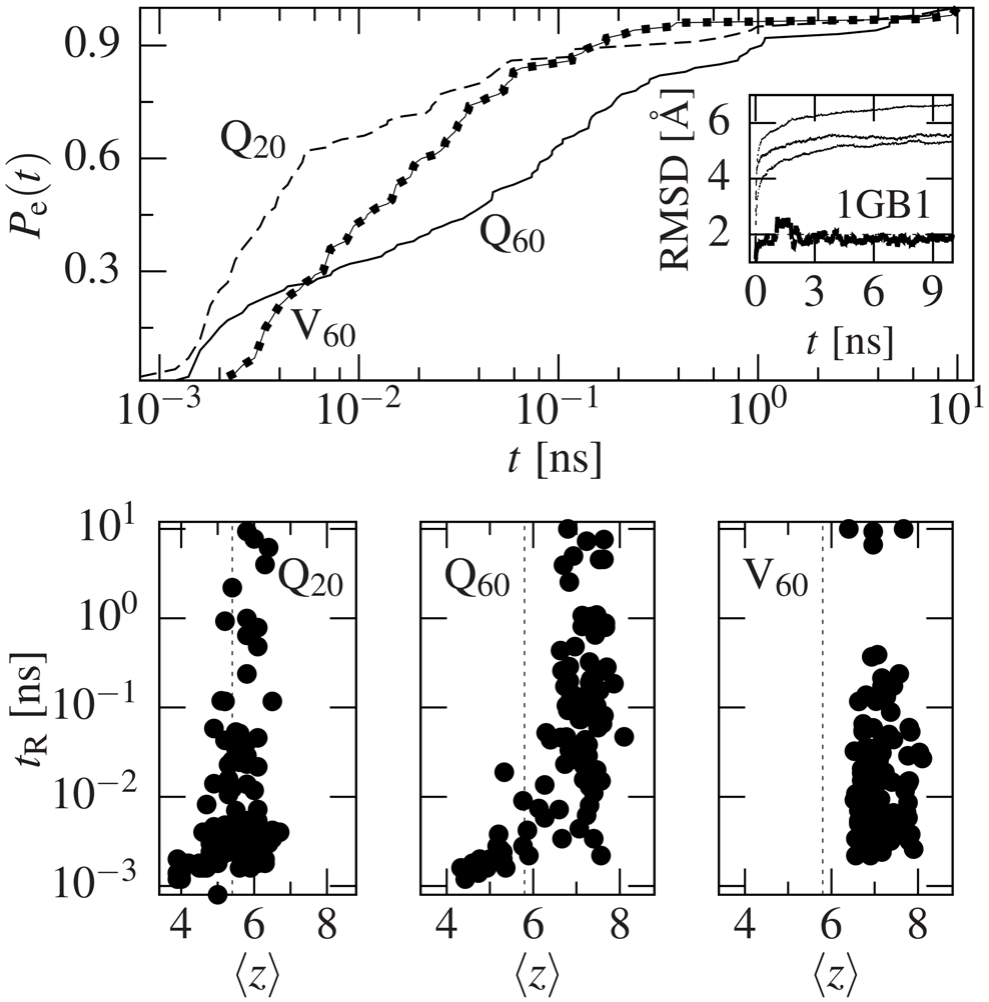
Time evolution of the studied structures. For each set in Q_60_, Q_20_ and V_60_, 100 randomly chosen structures have been placed under a free-dynamics evolution for 10 ns. After that, the RMSD has been studied and the last time when it fluctuates above 2 Å is recorded as the residence time (*t*_R_). The top graph shows the escape probability (*P*_e_(*t*)), defined as the probability of having left the initial state of a conformer at time *t*. We can see how Q_20_ fluctuates out of the initial structure much faster than Q_60_, while V_60_ starts more slowly but rapidly outruns both Q_60_ and Q_20_. The inset shows the average evolution of the RMSD for the three sets compared to an example of a similar-sized globular protein, an immunoglobulin binding domain of protein G (PDB code 1GB1, 56 residues). The latter lasts for longer than 10 ns fluctuating around 2 Å, while the other three rapidly evolve out of the initial structure. The bottom graphs show scatter plots of ⟨*z*⟩ *vs*. *t*_R_. No simple relation can be established between these two quantities above the stiff limit (dashed vertical lines), while below it residence times never exceed 1 ns.

Fig 3 shows the results of this study. The top panel shows that Q_60_ conformers last longer than Q_20_ in a specific state, while the average escape probability of V_60_ initially is lower but soon rises much faster than the other two sets. For completeness, we run the same study on three regular proteins: Trp-cage (PDB code 1L2Y, 20 residues long), an immunoglobulin binding domain of protein G (1GB1, 56 residues) and the 27th immunoglobulin domain of human cardiac titin (1TIT, 89 residues). All of them remained in the same conformation for longer than 10 ns: their RMSD was never higher than 2 Å.

The bottom panels of Fig 3 show scatter plots of *t*_R_ *vs*. ⟨*z*⟩. This figure shows that ⟨*z*⟩ is not only unrelated to *F*_max_, but also to the temporal stability of the conformers (as measured by *t*_R_) in the cases where ⟨*z*⟩ is above the simply stiff limit. In the case of conformers with ⟨*z*⟩ below this limit, however, *t*_R_ is always below 1 ns, reinforcing Maxwell’s theory on frame stiffness [35].

Interestingly, both theoretical and especially experimental pulling experiments are typically done at *v*_*p*_’s such that the time the protein is being pulled is far longer than 10 ns. In particular, the pulling simulations performed in this work take ≈ 50 *μ*s to completely extend a protein with 60 residues, while experiments such as the ones performed in [16] take around 60 ms to accomplish the same task. This leads to question whether the force peaks present in the experimental traces really relate to the initial conformers or have actually been formed while the molecule was being pulled.

Therefore, one must look at *F*_max_ carefully since it has different meaning in this kind of simulation than in experiments such as those in [16]: Here, mechanical stability is associated directly with a conformer, since simulations are based on the initial contact map. On the other hand, in experiments, molecules are subjected to fluctuations with a characteristic time of 1 ns and the *F*–*d* curves carry information not only about the initial conformer but also about the stretching-unrelated intrinsic shape transformations that the protein may undergo. All in all, we observe that disordered proteins such as polyglutamines are not long lasting when compared to structured globular ones, and that mechanical stabilities need to be looked at in the context of how they were measured, either referred to the initial conformer if done through structure-based modelling, or including bond formation during the stretching if performed experimentally.

### Structures with knots

Even though the starting structures were not knotted, our BEMD simulation yielded some knotted conformers. In particular, (9.3 ± 1.8)% of the independent Q_60_ conformers have a knot, while Q_20_ include no knotted conformers. Moreover, only (3.6 ± 0.5)% of the V_60_ structures contain a knot, and none of the CATH structures have one. All knots generated in V_60_ are trefoil (3_1_), while Q_60_ also contains one three-twist (5_2_). Upon stretching, only (13 ± 7)% of the Q_60_ knotted structures untie, while (45 ± 6)% of the V_60_ ones do. As shown in ref. [44], tightening of knots may be associated with force peaks. Both for Q_60_ and V_60_, knot tightening yields *F*_max_ from 0.9 to 2.4 *ɛ*/Å.

**Fig 4:**
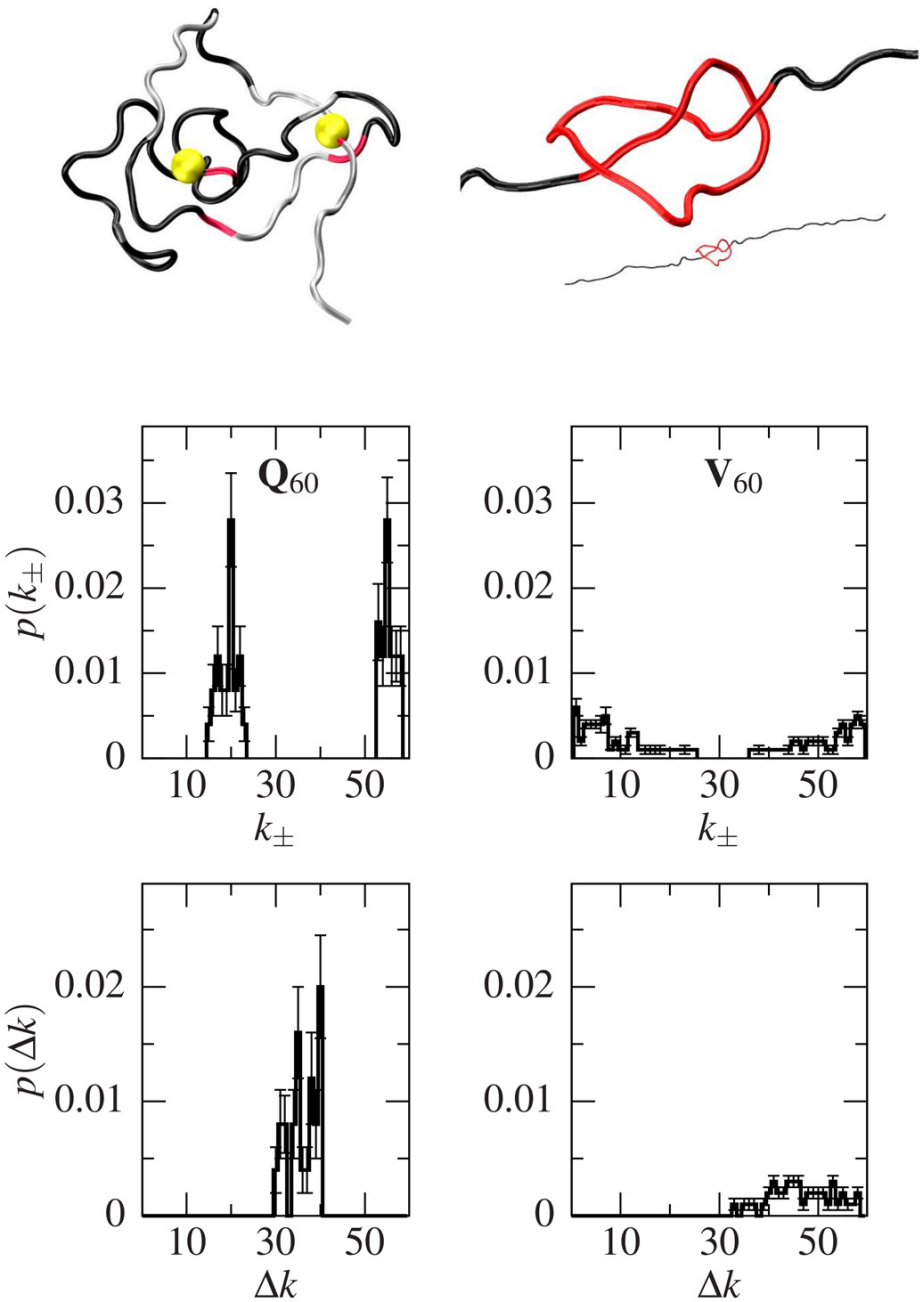
Knots in the studied conformers. The top left panel shows an example of a Q_60_ conformation containing a trefoil (3_1_) knot with the knot ends highlighted with yellow spheres. To its right, the same conformation has been partially stretched, and the region inside the knot is highlighted in red and zoomed in. The middle panels represent histograms of the knot end positions, *k*_±_, for Q_60_ (left) and V_60_ (right). The bottom panel shows their corresponding extension, Δ*k*. The percentage of knotted structures relative to to the total number of independent conformers found for Q_60_ and V_60_ are (9.3 ± 1.8)% and (3.6 ± 0.5)%, respectively. Shallow knots have an extension closer to 60 (the system size). Protein representations have been done with VMD [48].

Fig 4 shows an example of a 3_1_ knotted conformer found in Q_60_ plus a histogram of the ends of the knots (*k*_−_, *k*_+_) and their extension (Δ*k*, measured as the number of residues contained inside the knot) in the structures formed by sets Q_60_ and V_60_. An example of a 5_2_ one from Q_60_ and a 3_1_ from V_60_ are shown in S7 Fig Significantly, not only does V_60_ form fewer and less stable knots than Q_60_, but also the extension of the V_60_ knots is typically larger than the Q_60_ ones. Furthermore, the average extension of the knotted Q_60_ conformers is 36 (with a 0.12% error), which corresponds to the median threshold value for most polyglutamine-expansion-related diseases such as HD. We note that knotted structures would have been found experimentally as putative events in [16], since the final length would be reduced and thus they would render molecules with lower total contour length increase. Furthermore, knotted proteins have previously been found in nature especially in enzymes such as methyltransferases and carbonic anhydrases [45–47]. Nonetheless, the only hypothesized function of the knot itself—as opposed to the whole protein—is to prevent the unfolding of the protein in a case where the proteasome were to try to degrade it [46].

The presence of knots on its own is not indicative of their relevance: they need to last long enough to be able to have any effect. To that end, we performed 200 ns all-atom simulations with explicit TIP3P water of three randomly chosen knotted conformers to see the behaviour of these knots with time. The knot ends fluctuate along the protein as does the knot size, and in some cases the knot unties just to be formed again some time later –the time of the protein being in the untied conformation lasting for as long as 200 ps. Also, in two of the three cases, preferred places for the left and right ends can be seen, the right end being the same for both of them. The results of these simulations can be seen in S8 Fig.

### Other lengths in polyQ chains

Given that the average extension of the knots corresponds with the median of the threshold of the polyQ diseases, we applied the same methodology to other Q_*n*_ tracts, with *n* = 16, 25, 33, 38, 40 and 80. As expected, no knots were found for *n* < 35; but there were no knots in sets Q_38_, Q_40_ or Q_80_ either. This may be attributed to a low probability of knot formation combined with small statistics, which would imply that BEMD took Q_60_ through a knot-forming path while taking the rest of Q_*n*_ studied through non-forming ones. This is reinforced by the fact that the greater statistics of V_60_ do find knotted conformers. Therefore, an increase in the sampling may catch these knotted structures in Q_80_ and Q_40_, while their formation is fairly improbable for *n* below 35 since the typical knot size is about this length.

**Fig 5:**
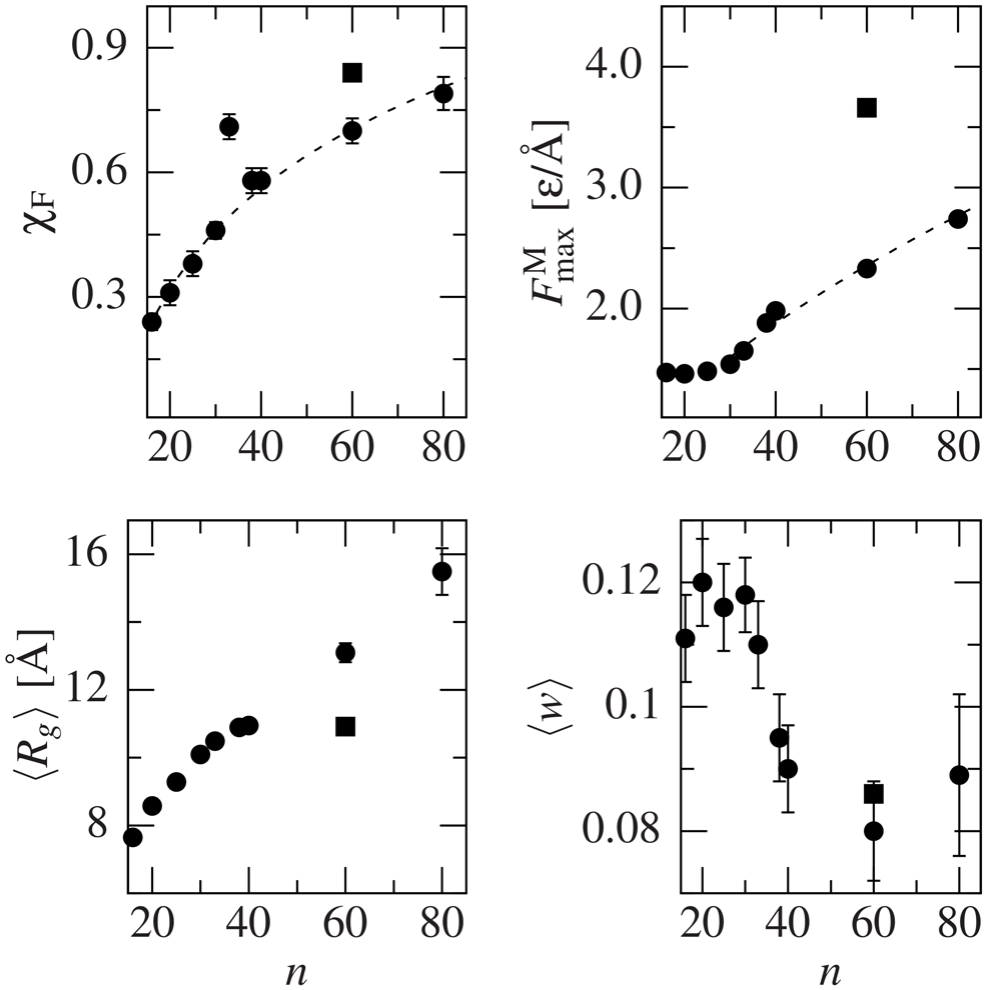
Variability of the specified parameters with the length, *n*, of the polyQ chain (circles). The values for V_60_ are indicated by a square. *χ*_*F*_ represents the fraction of conformers with at least one force peak for that particular length. The dotted fits correspond to a logarithmic function (top left.352 ln(*x*/8.115)) and a polynomial behavior (top right, *y* = 0.236*x* ^0.562^), which is typical for avalanches. The bottom panels show average over the structures of *R*_*g*_ and *w*. ⟨*R*_*g*_⟩ has a saturating behavior up to *n* = 40, but jumps for higher values. ⟨*w*⟩ presents a transition around *n* = 35 from slightly elongated to more globular proteins.

Fig 5 shows the evolution of the mechanical stability and shape with the chain length. In particular, the fraction of conformers with *F*_max_ > 0, which we name *χ*_F_, follow a logarithmic law, while the maximum *F*_max_ for each set, denoted as $F_{\text{max}}^{\text{M}}$ behaves like an avalanche system: it has a constant value until *n* = 33, and then starts growing as a power law with exponent 0.562. The average *R*_*g*_ appears to be saturating as *n* approaches 40, but it suddenly jumps for *n* = 60 and 80. Judging by *w*, the shapes of the conformers change around *n* = 35 from elongated to more globular. Interestingly, V_60_ behaves differently than Q_60_ except that the average *w* (lower right panel) is similar, suggesting the similarity of shapes. We also conclude that the fraction of mechanically stable conformers increases uniformly with *n*, while the maximum *F*_max_ presents an avalanche behaviour for *n* > 30, once again close to HD’s threshold of 35.

## Discussion

In this study, we have generated an ensemble of structurally independent conformers for glutamine expansions with *n* residues. We have focused on *n* = 60 which, if present in huntingtin protein, would result in Huntington disease, and on *n* = 20, which would not.

We have then expanded the study to *n* = 16, 25, 30, 33, 38, 40 and 80 in order to further explore the structural nature of the *n* ≈ 35 threshold in most polyQ-related diseases.

We find that proteins related to the disease exhibit less conformational polymorphism than the ones unrelated to it in terms of independent structures and transition kinetics, even though the former show much more mechanical variability (in terms of *F*_max_ and *n*_p_) as well as structural (measured by SS and ⟨*z*⟩). We also conclude that, contrary to intuition, ⟨*z*⟩, *CO*, SS and *β*-content are not good predictors of either temporal or mechanical stability. This conclusion extends not only for polyQ but also generally for all proteins in CATH.

Finally, we prove the presence of knots of length 35 at least in Q_60_. The sequential size of these knots suggests a relationship to HD. One of the possible mechanisms for the relevance of the knots in pathology is impairing the process of proteasomal degradation, as suggested in [46] and [16]. Moreover, although there is evidence for the toxicity of the monomeric polyQ species [49], even if the toxicity was due mainly to the oligomers (see *e.g*. Ref. [50]), the blockade of the degradation machinery by a knotted monomer would induce an increase of the concentration of aggregating protein, and thus toxicity may be caused by the monomers even if they are not toxic themselves.

## Supporting Information

S1 Text The details of structure generation and selection are explained here, together with the stability associated to the coordination number. Furthermore, a statistical analysis of the lack of relation between *F*_max_ and the rest of the descriptors used in this work is also presented.
(PDF)

S1 Fig Five conformers with highest mechanical stability in set Q_60_. The structure with the biggest *F*_max_ is at the top. The left column shows snapshots of the structures. The red ribbons represent β strands and the red lines correspond to β bridges. The black lines indicate hydrogen-bonded turns. The orange spheres mark the termini, from which the molecule is pulled. The center column displays the unfolding *F* − *d* curve (left axis) together with the unfolding scenario diagram (right axis), *i.e*. the time a contact is broken *vs*. the distance between the residues that are in contact. The column on the right shows the values of the relevant descriptors. All molecule cartoons were generated using VMD [48].
(TIF)

S2 Fig Five conformers with highest mechanical stability in set Q_20_. The structure with the biggest *F*_max_ is at the top. The left column shows snapshots of the structures. The red ribbons represent β strands and the red lines correspond to β bridges, while blue helices are α helices. The black lines indicate hydrogen-bonded turns. The center column displays the unfolding *F* − *d* curve (left axis) together with the unfolding scenario diagram (right axis). The column on the right shows the values of the relevant descriptors.
(TIF)

S3 Fig Five conformers with highest mechanical stability in set V_60_. The structure with the biggest *F*_max_ is at the top. The left column shows snapshots of the structures. The red ribbons represent β strands and the red lines correspond to β bridges. The black lines indicate hydrogen-bonded turns and α-helices are depicted in blue. The center column displays the unfolding *F* − *d* curve (left axis) together with the unfolding scenario diagram (right axis). The column on the right shows the values of the relevant descriptors.
(TIF)

S4 Fig Scatter plot of *F*_max_ *vs*. α, β and hydrogen-bonded turns (τ) content for polyQ chains. The horizontal dashed lines mark off the top five values of *F*_max_.
(TIF)

S5 Fig Scatter plot of *F*_max_ *vs*. *CO* for the specified sets. The horizontal dashed lines mark off the top five values of *F*_max_.
(TIF)

S6 Fig Kinetics of independent structure formation for Q_60_ (circles), V_60_ (triangles) and Q_20_ (diamonds). Although more complete plots should be fit with a double exponential function [4], short trajectories correspond to a linear behavior. The fitted slopes are .28, .62 and .98 respectively. Data for V_60_ were taken from [4].
(TIF)

S7 Fig Examples of knotted structures. The top structure corresponds to a three-twist (5_2_) knot in Q_60_, while the lower panels are for a trefoil knot from V_60_, where no other knots were found. Left column shows a representation of the molecule before stretching, with the knot ends highlighted with yellow spheres. Right panels show the molecules partially stretched, and the region inside the knot is highlighted in red and zoomed in.
(TIF)

S8 Fig Time evolution of the knots. Three randomly chosen knotted conformers were simulated with all-atom and explicit solvent. One of them is shown in Fig 4. The top panel shows the evolution of the knot size with time for one of the simulations. The middle panel shows a histogram of the knot sizes along this time for the three simulations, each with a different color. The bottom panel shows a histogram of the respective knot ends, the left end (*k*_−_, inverted) and the right ones (*k*_+_).
(TIF)

S9 FigAn example of the SS sieve and time clustering stages.The gray line in the top panel shows evolution of SS with time for one of the replicas. Structures with SS > 30% (the thin horizontal line) are taken for clustering. A cluster ends whenever the gap between successive structured conformers becomes greater than 50 ps. The black dots correspond to structures that represent clusters: these are the structures with the highest SS in the cluster. The red box in the top panel is shown zoomed in the middle panel, where clusters are represented by red lines. The bottom panel shows the RMSD of each cluster representative relative to the previous one. All of these RMSD’s are greater than 2 Å so the clusters can be considered to be uncorrelated in time.(TIF)

S10 FigColor-map plots of the difference between the joint CDF and the product of the independent CDFs of *F*_max_ and the specified descriptor.Differences are always below 0.1, and below 0.05 in three of the descriptors (SS, *CO* and τ). Therefore, *F*_max_ is statistically independent of the descriptors studied.(TIF)

S1 Table Parameters of a linear regression for the dependence of *F*_max_ on various structural descriptors. The top panel lists the values of the Pearson *R* ^2^ coefficients with a 95% confidence interval. The lower panel lists the slopes of the linear fits together with the error bars. The number in the parenthesis is the corresponding *p*-value. Even though the slope for each correlation is significantly different from zero; *R* ^2^ is never close to one, so no correlation can be established between the descriptors and *F*_max_. This is also assessed in S10 Fig.
(PDF)
