# Supplementary material for: An Exploration of the Universe of Polyglutamine Structures
Source: PLoS Comput Biol. 2015 Oct 23;11(10):e1004541. doi: 10.1371/journal.pcbi.1004541 (PMC4619799; doi:10.1371/journal.pcbi.1004541)
Supplement: S1 Text — Furthermore, a statistical analysis of the lack of relation between F max and the rest of the descriptors used in this work is also presented. (PDF) [file pcbi.1004541.s001.pdf]

An Exploration of the Universe of Polyglutamine Structures - Submission to PLOS Journals

Àngel Gómez-Sicilia[1,2], Mateusz Sikora[3], Marek Cieplak[4,*], Mariano Carrión-Vázquez[1,2]

1 Intituto Cajal/CSIC, Avda. Doctor Arce 37, 28002 Madrid (Spain)
2 Instituto Madrileño de Estudios Avanzados en Nanociencia (IMDEA-Nanociencia), C/ Faraday 9, 28049 Cantoblanco, Madrid (Spain)
3 Institute of Science and Technology Austria, Am Campus 1, 3400 Klosterneuburg (Austria)
4 Instytut Fizyki PAN, Al. Lotników 32/46, 02668 Warsaw (Poland)

*** mc@ifpan.edu.pl**

Generation of the independent structures

The BEMD technique is similar in spirit to the replica exchange method combined with metadynamics. In the former, several replicas of the system are simulated at different temperatures and each system is allowed to switch from one temperature to another with the Boltzmann probability. The latter keeps a memory of the visited states of particular collective variables (*e.g.* Ramachandran angles, hydrogen bonds or α-structured regions) and biases the system to explore unvisited states.

The BEMD, however, instead of switching between different temperatures, it involves switching between the collective variables that are selected for biasing. In particular, we use three collective variables: α-helix RMSD, antiparallel β-strand RMSD and parallel β-strand RMSD, where RMSD is measured with respect to small ideal structures. Ideal structures are defined as those calculated as an average of secondary structure motifs found in experimental structures. Thus, every 6-residue segment is compared to an ideal α-helix, and every pair of 3-residue segments is compared to an ideal parallel or antiparallel β-strand. A more detailed explanation of the collective variables can be found in [1].

Our system is simulated with the use of six replicas, where one is unbiased; three of them have an α bias, each on a different third of the protein sequence; and the other two have antiparallel and parallel β biases throughout the whole sequence. The simulations are run using implicit water, with an integration step of 0.2 fs, and at a temperature of $T = 400\ K$ (controlled by the Nosé-Hoover thermostat). The higher temperature helps a faster exploration of the energy landscape [2]. The biases are added to the potential in the form of the Gaussian functions of height 20.92 kJ/mol and width 0.3 nm every 10 ps, and exchanges between biases are allowed every 25 ps. We save the coordinates of all atoms in the system every 5 ps, which generates a snapshot.

For computational efficiency, it is better to keep a constant bias in every replica and transfer the atom coordinates from one to another, instead of having the atom coordinates fixed in a replica and switching the biases. Thus, the snapshots we obtain from the simulation are not continuous with respect to time, but are attached to the particular bias, since each set of the coordinates jumps around every few steps. In order to recover time-ordered trajectories, we use our own script.

Once the snapshots are properly ordered in time we obtain six trajectories with a snapshot taken every 5 ps. At this stage, a three-sieve method is applied in order to

obtain structures that are temporally and structurally independent. In the first step, the DSSP program is used to obtain the $\mathbb{SS}$ content for each snapshot. Those with $\mathbb{SS} < 30$ % are discarded, while the rest are considered structured and forwarded to the second sieve.

The second sieve is used to find temporally-uncorrelated structures from those obtained in the first stage. To this end, we select the end of a time cluster to be at the point where two structured conformers are separated by at least 50 ps of unstructured ones. The conformer in each cluster with higher $\mathbb{SS}$ is chosen to represent the cluster and proceeds to the third and final sieve. S9 Fig. presents an example of the first and second sieve of one of the replicas. In the top panel, the temporal evolution of $\mathbb{SS}$ is shown together with the cluster representatives. The red box marks a 50 ns time frame which is then expanded in the middle panel. In this panel, the clusters obtained after the second sieve in this time frame are also marked with a red line. The bottom panel shows that each representative is not correlated with its predecessor, since their mutual RMSD is always greater than 2 Å [3].

The third sieve checks for the structural independence, and is carried out on all time-cluster representatives irrespective of the replica they originate from. Conformers coming from the second sieve are classified according to their structural independence. In order to study this feature, we use TM-score [4] and our version of the TM-align algorithm [5] in which the determination of the secondary structure is based on the results coming from DSSP. Cossio *et. al.* [2] have shown the modified version to perform better.

TM-score is a value that measures similarity between two proteins according to an atomic alignment, which can be based either on sequence or secondary structure. In this case, sequential alignment is not needed since all conformers contain the same sequence. After the alignment is done, only the aligned atoms are taken into account by summing the inverse of the distances between them, then normalizing to the total length of the protein, as in equation 1, where n_a is the number of aligned atoms, n is the length of the protein, d_i is the distance between the atoms in the i-th pair and d_0 is a standard distance used for normalization. The max function refers to all possible alignments.

$$TM = \max\left[\frac{1}{n}\sum_{n_a}\frac{1}{1+\left(\frac{d_i}{d_0}\right)^2}\right] \quad (1)$$

Therefore, the TM-score would be 100 % if all the atoms in the proteins could be aligned with one another, and in this alignment they all were at the same position (meaning the distance between them would be 0). This process results in a matrix of scores that rate their similarity in a pair-wise fashion. As in ref. [2], two conformers are considered neighbors if their TM-score is greater than 45 %.

At this stage, we identify the conformation which has the highest number of neighbors. This conformation and its neighbors are denoted as a cluster, which is removed from the pool. The conformer in the cluster with the largest $\mathbb{SS}$ is selected to belong to the final set of structures. This procedure is then repeated with the conformations still remaining in the pool, until the pool becomes empty.

Therefore, after the three sieves, the structures obtained are temporally uncorrelated and structurally independent. For the studied sets of Q_n with $n = 16$, 20, 25, 30, 33, 38, 40, 60 and 80, as well as for V_{60}, the independent conformers can be found in `www.ifpan.edu.pl/~cieplak/POLYQ`.

The simply stiff limit of the average coordination number

Following the terminology used by Maxwell, we define an n-particle system with pairwise interactions to be *stiff* if it contains no pairs of particles that can be moved apart without affecting a bond. Furthermore, a stiff system is considered *simply stiff* if the removal of any bond will turn it into being not stiff.

The *coordination number* (z) of a particle is defined as the number of bonds it establishes with others.

The stiffness of the n-particle system depends on the dimensionality (D) of space in which the particles are set and on the number of bonds (b) between them. Instead of b, we can discuss the dependence on the average coordination number because the two quantities are closely related. It is easy to see that the sum of the coordination numbers of all of the particles in the system is equal to the double of the number of bonds. This is because each bond connects two particles and thus it counts twice. Thus, the average coordination number for the system is given by

$$\langle z \rangle = \frac{2b}{n} \tag{2}$$

For particles moving along a line (one-dimensional system, one degree of freedom), a system is simply stiff if $b = n - 1$. This equation can be proved by using the method of mathematical induction. For $n = 2$, the system is simply stiff when one bond is present. If we have a simply stiff system of n particles and we add one more, then only one extra bond is needed to ensure that this new particle will not be able to move away from any other. Therefore, using eq. 2, the average coordination number for a simply stiff 1D system is

$$\langle z \rangle_{1\mathrm{D}} = 2 - \frac{2}{n} \tag{3}$$

For 2D and 3D systems, the number of bonds in a simply stiff particle system is $b = 2n - 3$ and $b = 3n - 6$, respectively. The proof can be obtained in analogy to the 1D case. Therefore, the average threshold coordination number is given by

$$\langle z \rangle_{2\mathrm{D}} = 4 - \frac{6}{n} \tag{4}$$

$$\langle z \rangle_{3\mathrm{D}} = 6 - \frac{12}{n} \tag{5}$$

In the thermodynamic limit ($n \to \infty$) of a 3D system the threshold $\langle z \rangle$ is 6, as shown by Maxwell. For finite protein-like systems, this threshold value is reduced. In the cases of this study, simply stiff systems should correspond to $\langle z \rangle = 5.4$ if n is 20 and $\langle z \rangle = 5.8$ if n is 60.

Lack of dependence of $F_{\mathbf{max}}$ on the structural descriptors

To establish the degree of correlation between $F_{\max}$ and the the structural descriptors, we performed a linear fit of the data as shown shown in various plots throughout the paper. The combined results on the slopes and the Pearson coefficients are shown in S1 Tab. We conclude that even if a trend could be established given the non-zero slope coefficients, a linear relation cannot be established since Pearson's R^2 coefficient is never sufficiently close to 1. Nonetheless, statistical independence between two events cannot be inferred simply from the scatter plot.

Indeed, lack of correlation –*i.e.* the data cannot be fitted with a straight line– does not rule out the possibility that other (non-linear) relations could be established. In

order to figure out whether the $F_{\max}$ is indeed independent of the structural descriptors used here, we performed another statistical analysis by applying the definition of statistical independence: Two random variables X and Y, distributed according to the cumulative density functions (CDF) $F_X(x)$ and $F_Y(y)$, respectively, are independent if and only if $F_X(x) \cdot F_Y(y) = F_{X,Y}(x, y)$.

To this end, we studied the joint CDF of $F_{\max}$ with each of the other descriptors and compared it to the product of the independent probabilities. S10 Fig. depicts the results of this study for CATH in the form of the absolute difference between each pair of two-dimensional distributions. The rest of the sets are not shown but the results are equivalent. We can observe that the difference between the two is below 5 % for $\mathbb{SS}$, CO and τ; and below 10 % for the rest. Thus, we assume the CDFs to be approximately equal and thus $F_{\max}$ to be independent of the descriptors.

References

1. Pietrucci F, Laio A. A Collective Variable for the Efficient Exploration of Protein Beta-Sheet Structures: Application to SH3 and GB1. J Chem Theory Comput. 2009;5(9):2197–2201. Available from: http://dx.doi.org/10.1021/ct900202f.

2. Cossio P, Trovato A, Pietrucci F, Seno F, Maritan A, Laio A. Exploring the universe of protein structures beyond the Protein Data Bank. PLoS Comput Biol. 2010;6(11):e1000957. Available from: http://dx.doi.org/10.1371/journal.pcbi.1000957.

3. Piana S, Laio A. A bias-exchange approach to protein folding. The J Phys Chem B. 2007 May;111(17):4553–4559. Available from: http://dx.doi.org/10.1021/jp067873l.

4. Zhang Y, Skolnick J. Scoring function for automated assessment of protein structure template quality. Proteins: Struct Funct Bioinforma. 2004;57(4):702–710. Available from: http://dx.doi.org/10.1002/prot.20264.

5. Zhang Y, Skolnick J. TM-align: a protein structure alignment algorithm based on the TM-score. Nucleic Acids Res. 2005;33(7):2302–2309. Available from: http://dx.doi.org/10.1093/nar/gki524.
