# Supplementary figures and images for: An Exploration of the Universe of Polyglutamine Structures
Source: PLoS Comput Biol. 2015 Oct 23;11(10):e1004541. doi: 10.1371/journal.pcbi.1004541 (PMC4619799; doi:10.1371/journal.pcbi.1004541)

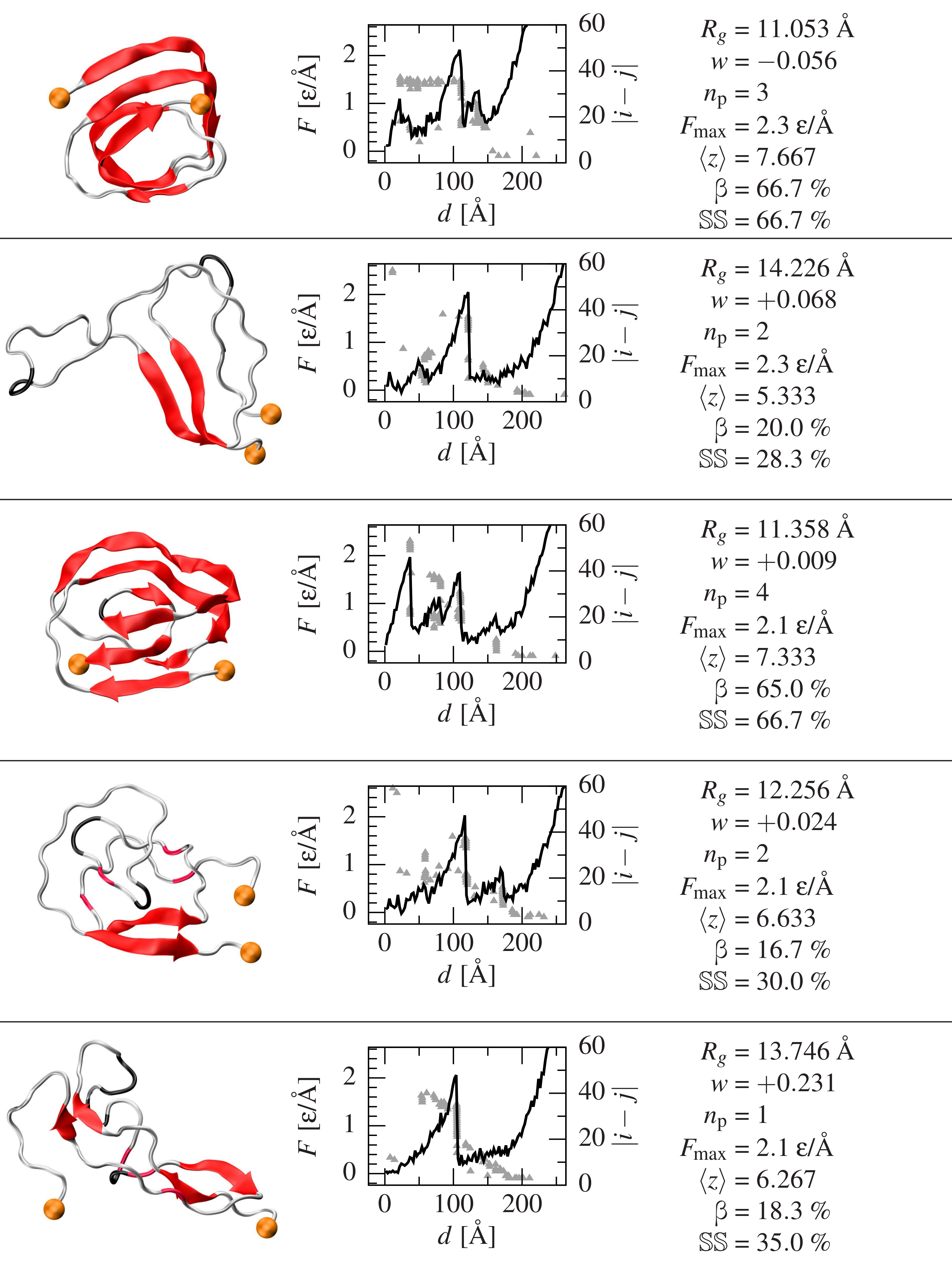

Supplement: S1 Fig — The structure with the biggest F max is at the top. The left column shows snapshots of the structures. The red ribbons represent β strands and the red lines correspond to β bridges. The black lines indicate hydrogen-bonded turns. The orange spheres mark the termini, from which the molecule is pulled. The center column displays the unfolding F − d curve (left axis) together with the unfolding scenario diagram (right axis), i.e. the time a contact is broken vs. the distance between the residues that are in contact. The column on the right shows the values of the relevant descriptors. All molecule cartoons were generated using VMD [48]. (TIF) [file pcbi.1004541.s002.tif]

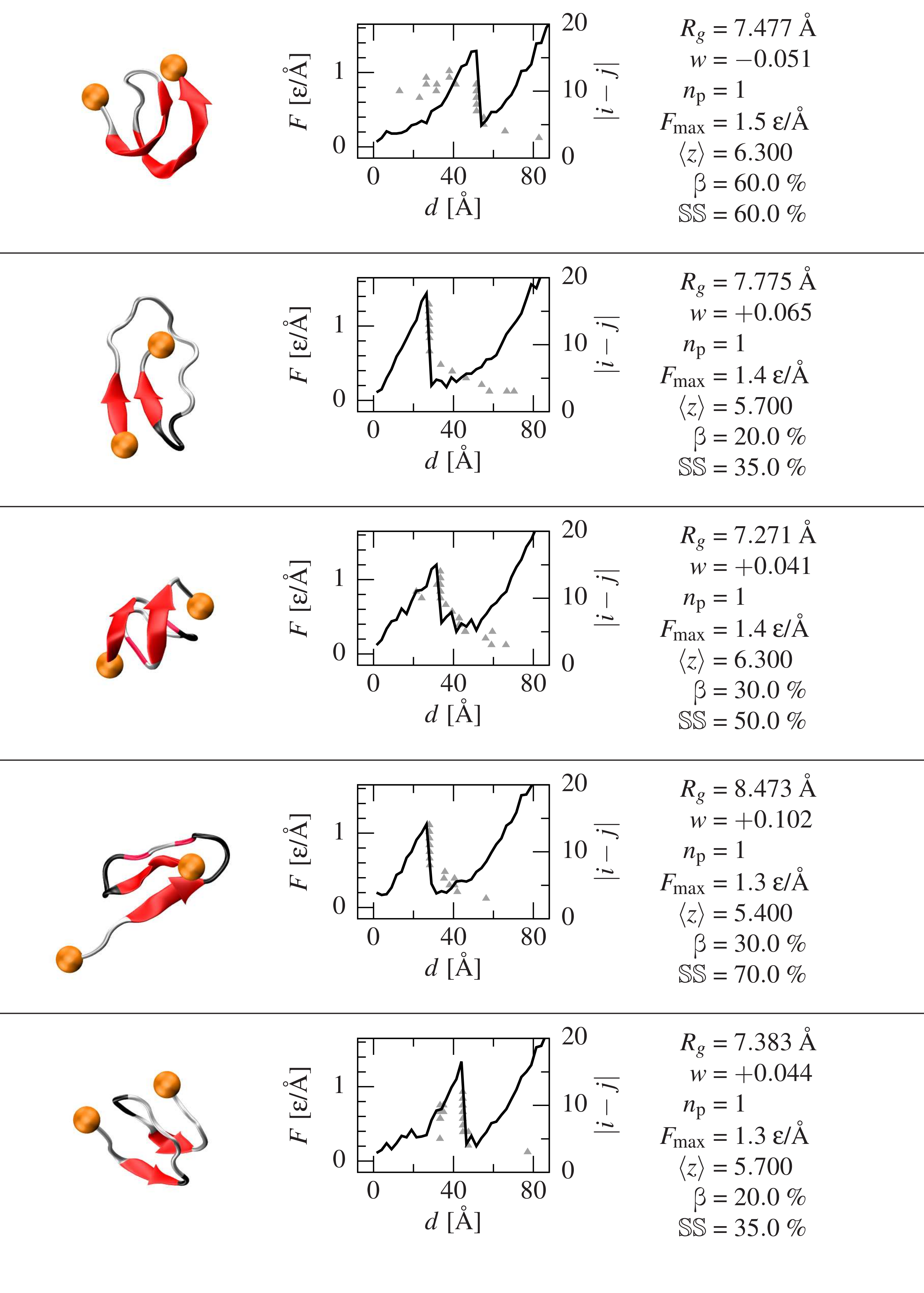

Supplement: S2 Fig — The structure with the biggest F max is at the top. The left column shows snapshots of the structures. The red ribbons represent β strands and the red lines correspond to β bridges, while blue helices are α helices. The black lines indicate hydrogen-bonded turns. The center column displays the unfolding F − d curve (left axis) together with the unfolding scenario diagram (right axis). The column on the right shows the values of the relevant descriptors. (TIF) [file pcbi.1004541.s003.tif]

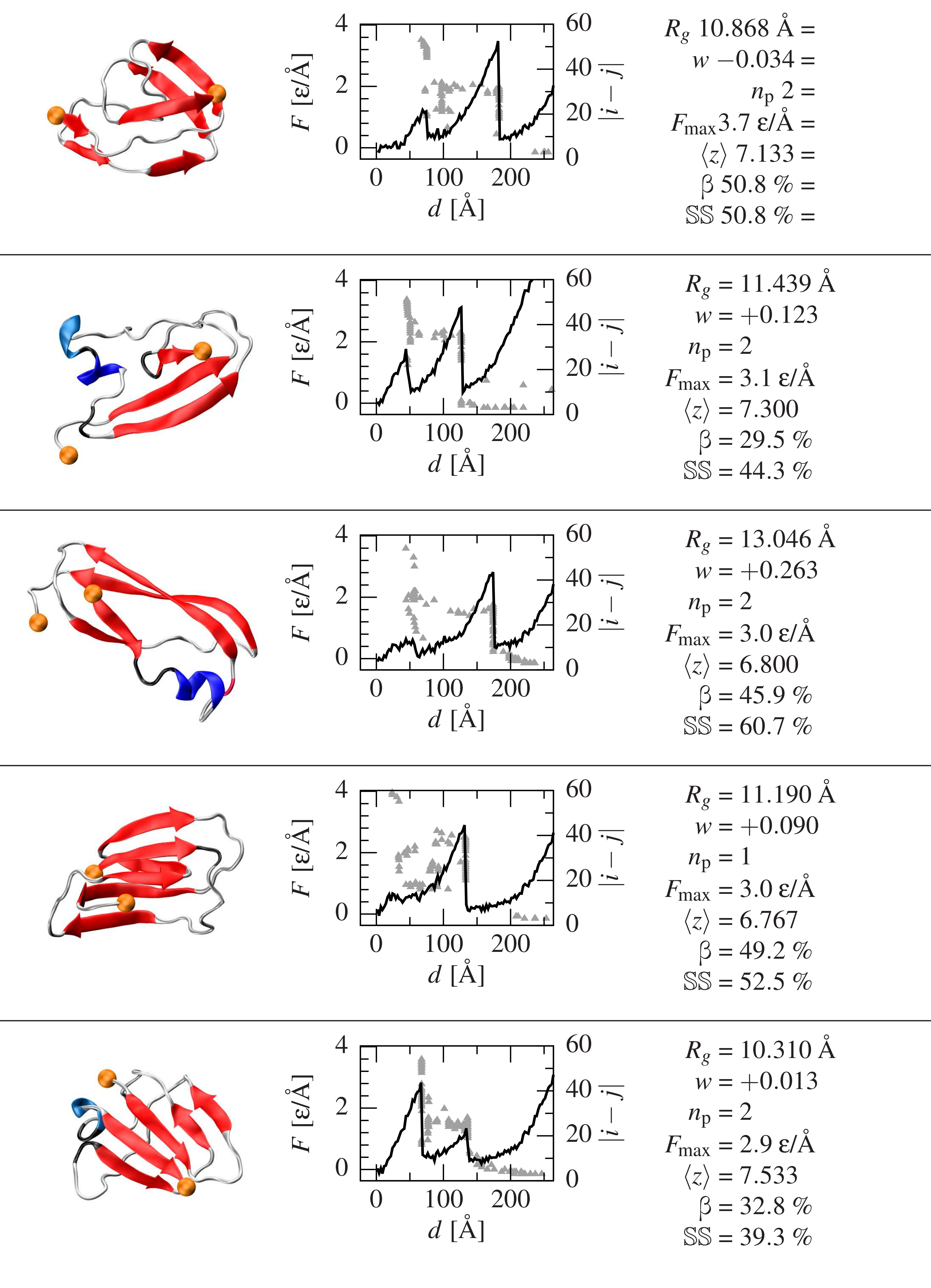

Supplement: S3 Fig — The structure with the biggest F max is at the top. The left column shows snapshots of the structures. The red ribbons represent β strands and the red lines correspond to β bridges. The black lines indicate hydrogen-bonded turns and α-helices are depicted in blue. The center column displays the unfolding F − d curve (left axis) together with the unfolding scenario diagram (right axis). The column on the right shows the values of the relevant descriptors. (TIF) [file pcbi.1004541.s004.tif]

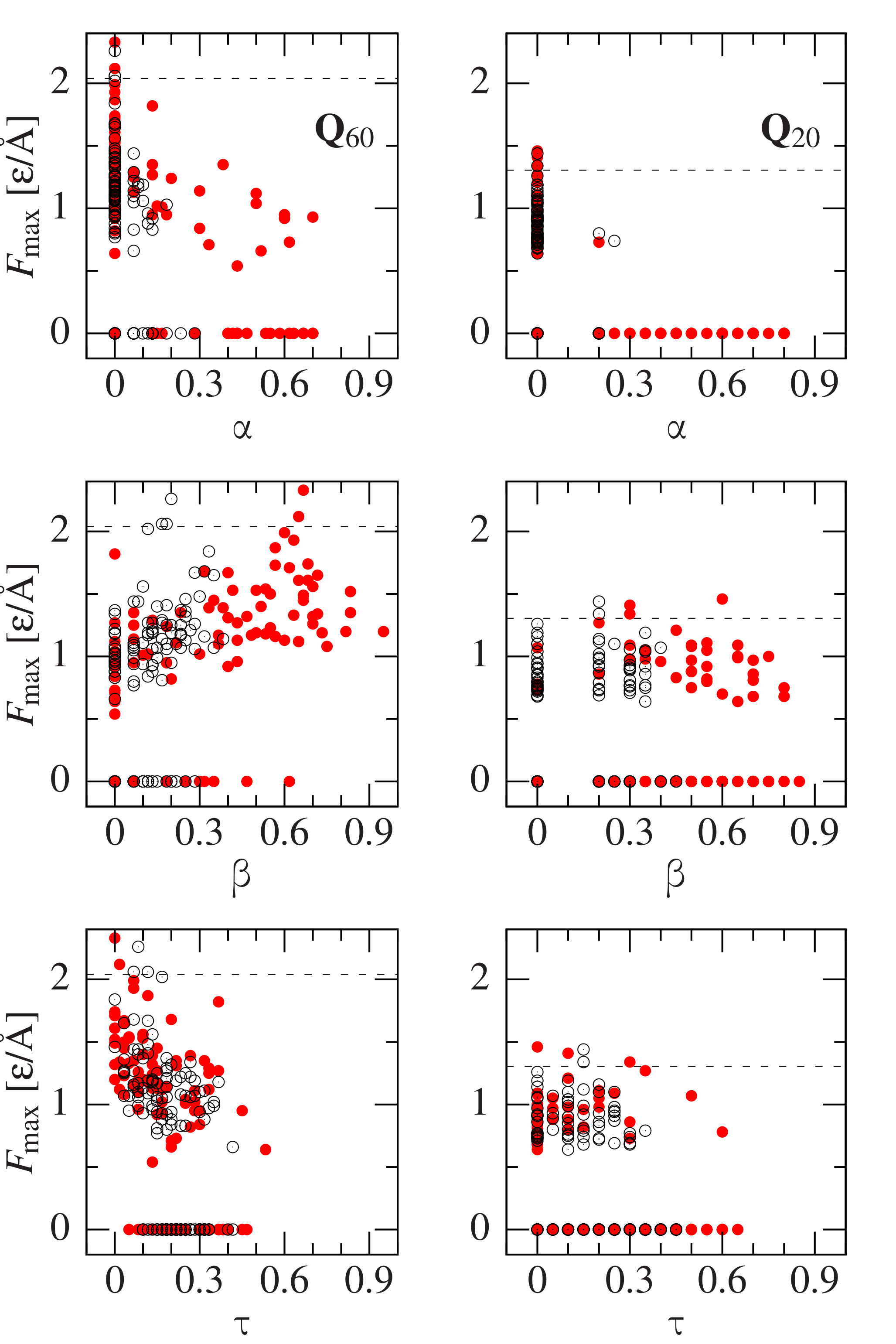

Supplement: S4 Fig — The horizontal dashed lines mark off the top five values of F max. (TIF) [file pcbi.1004541.s005.tif]

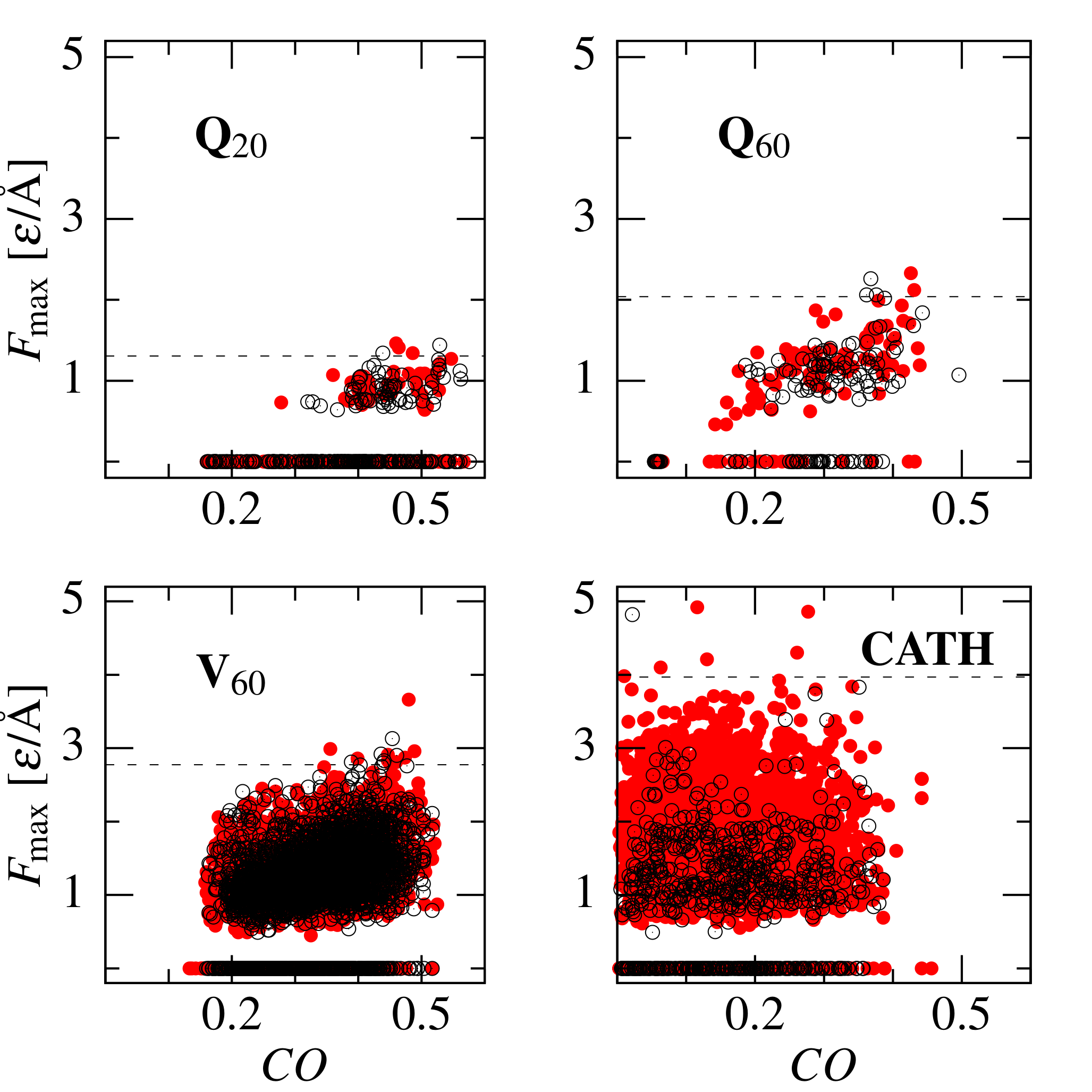

Supplement: S5 Fig — The horizontal dashed lines mark off the top five values of F max. (TIF) [file pcbi.1004541.s006.tif]

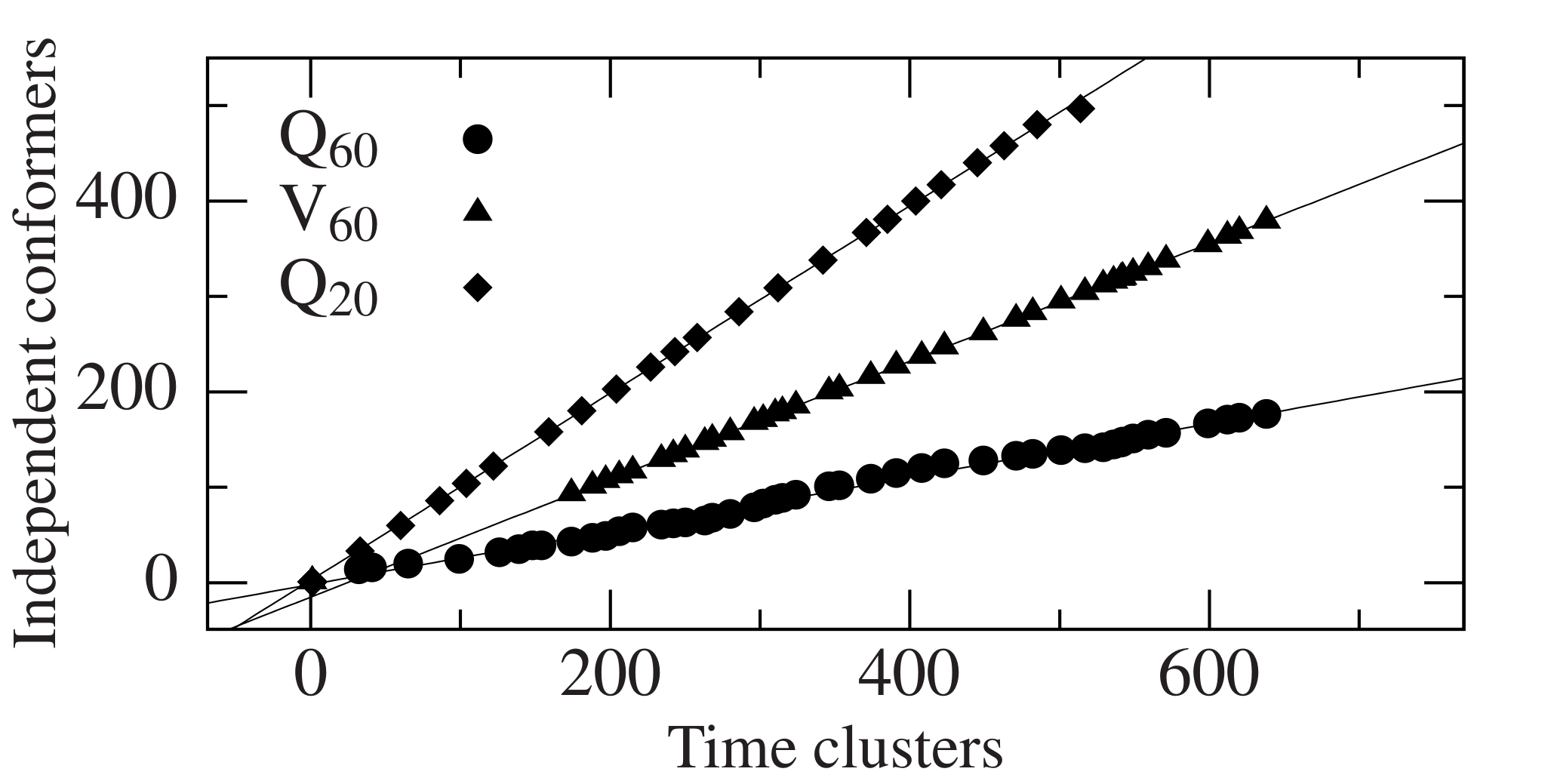

Supplement: S6 Fig — Although more complete plots should be fit with a double exponential function [4], short trajectories correspond to a linear behavior. The fitted slopes are .28, .62 and .98 respectively. Data for V60 were taken from [4]. (TIF) [file pcbi.1004541.s007.tif]

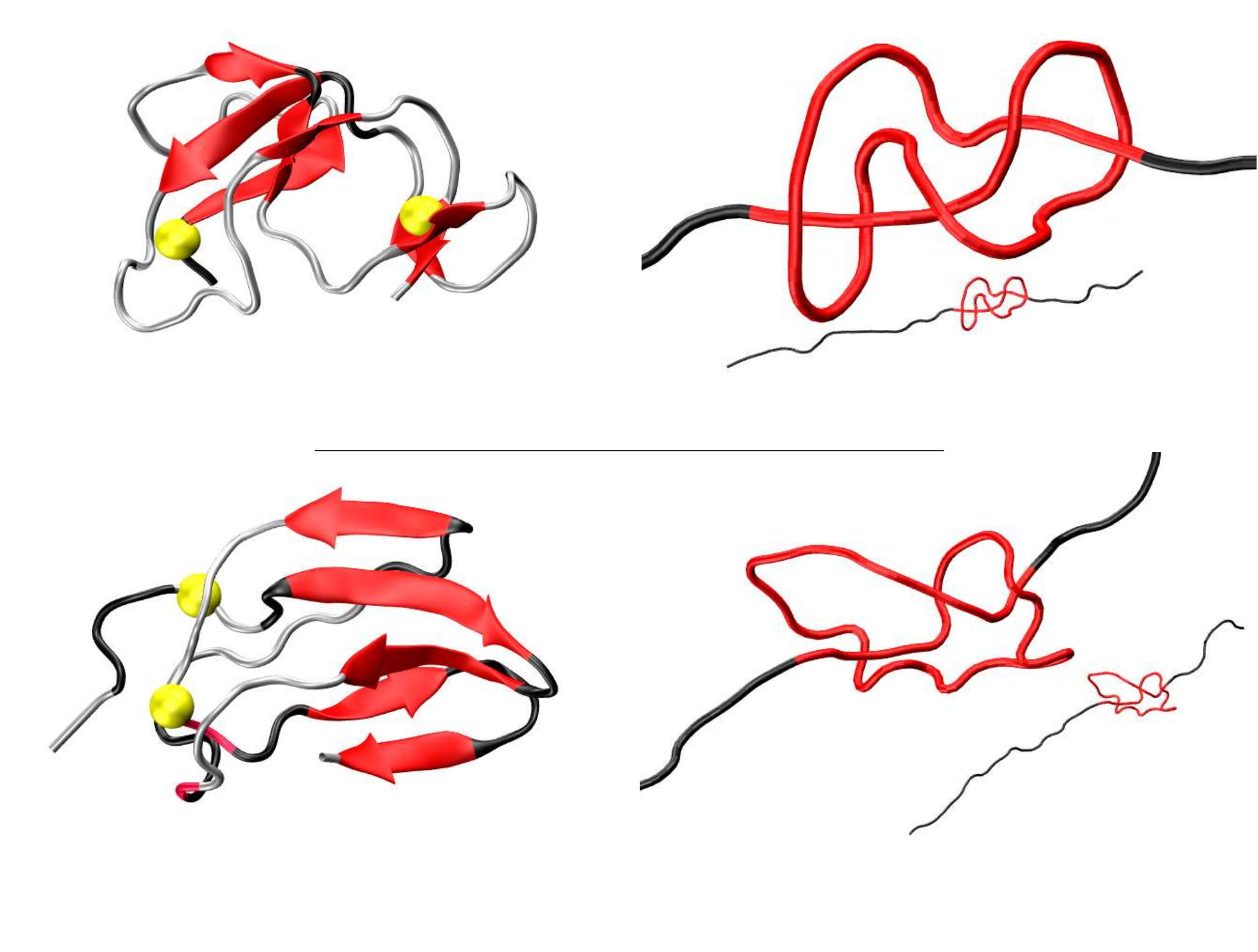

Supplement: S7 Fig — The top structure corresponds to a three-twist (52) knot in Q60, while the lower panels are for a trefoil knot from V60, where no other knots were found. Left column shows a representation of the molecule before stretching, with the knot ends highlighted with yellow spheres. Right panels show the molecules partially stretched, and the region inside the knot is highlighted in red and zoomed in. (TIF) [file pcbi.1004541.s008.tif]

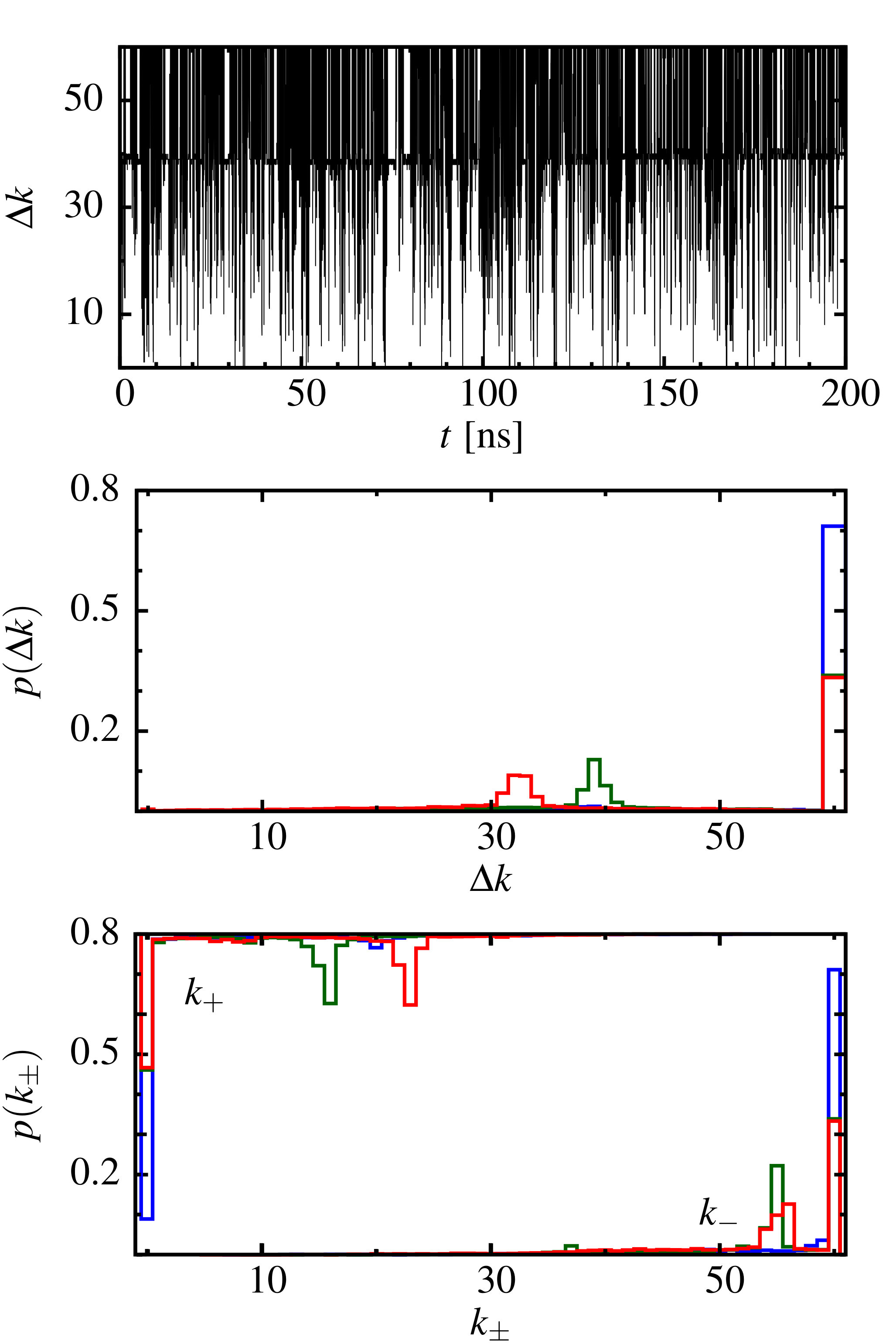

Supplement: S8 Fig — Three randomly chosen knotted conformers were simulated with all-atom and explicit solvent. One of them is shown in Fig 4. The top panel shows the evolution of the knot size with time for one of the simulations. The middle panel shows a histogram of the knot sizes along this time for the three simulations, each with a different color. The bottom panel shows a histogram of the respective knot ends, the left end (k −, inverted) and the right ones (k +). (TIF) [file pcbi.1004541.s009.tif]

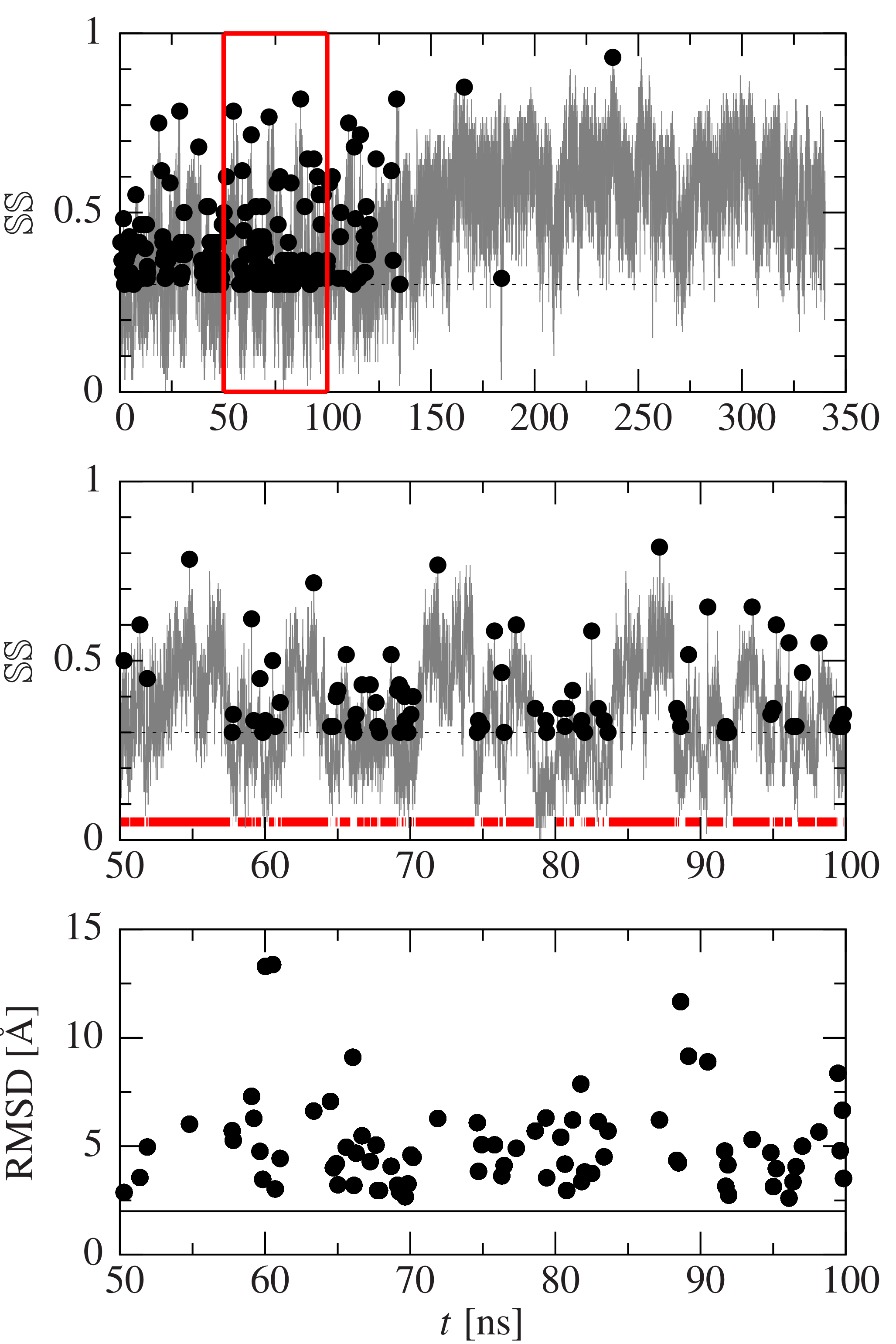

Supplement: S9 Fig — The gray line in the top panel shows evolution of SS with time for one of the replicas. Structures with SS > 30% (the thin horizontal line) are taken for clustering. A cluster ends whenever the gap between successive structured conformers becomes greater than 50 ps. The black dots correspond to structures that represent clusters: these are the structures with the highest SS in the cluster. The red box in the top panel is shown zoomed in the middle panel, where clusters are represented by red lines. The bottom panel shows the RMSD of each cluster representative relative to the previous one. All of these RMSD’s are greater than 2 Å so the clusters can be considered to be uncorrelated in time. (TIF) [file pcbi.1004541.s010.tif]

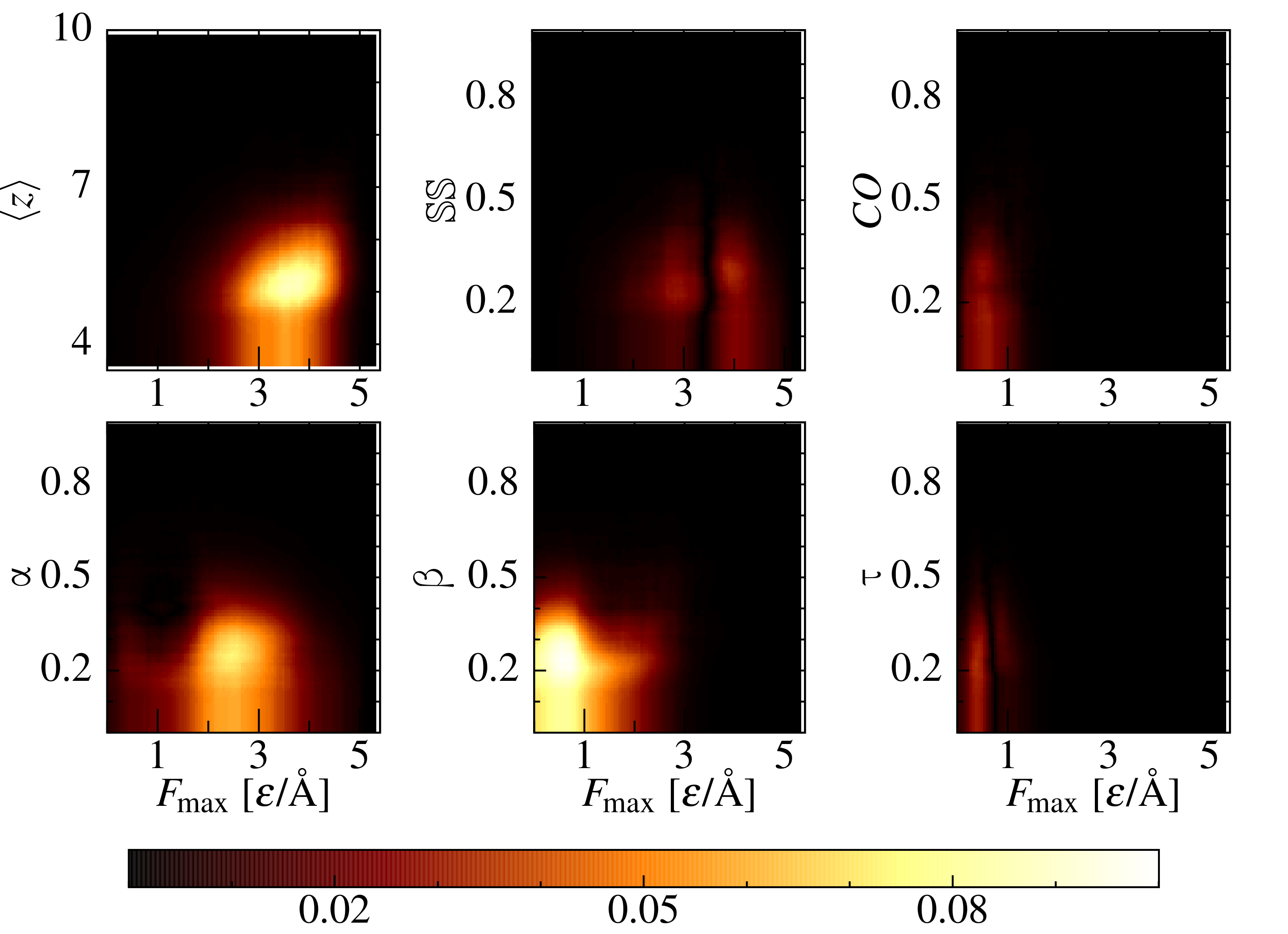

Supplement: S10 Fig — Differences are always below 0.1, and below 0.05 in three of the descriptors (SS, CO and τ). Therefore, F max is statistically independent of the descriptors studied. (TIF) [file pcbi.1004541.s011.tif]
