# Supplementary material for: An Exploration of the Universe of Polyglutamine Structures
Source: PLoS Comput Biol. 2015 Oct 23;11(10):e1004541. doi: 10.1371/journal.pcbi.1004541 (PMC4619799; doi:10.1371/journal.pcbi.1004541)
Supplement: S1 Table — The top panel lists the values of the Pearson R 2 coefficients with a 95% confidence interval. The lower panel lists the slopes of the linear fits together with the error bars. The number in the parenthesis is the corresponding p-value. Even though the slope for each correlation is significantly different from zero; R 2 is never close to one, so no correlation can be established between the descriptors and F max. This is also assessed in S10 Fig. (PDF) [file pcbi.1004541.s012.pdf]

R^2

	Q_{20}	Q_{60}	V_{60}	CATH
$\langle z \rangle$	0.09 ± 0.05	0.14 ± 0.08	0.064 ± 0.011	0.21 ± 0.02
CO	0.11 ± 0.05	0.34 ± 0.10	0.119 ± 0.014	0.021 ± 0.007
$\mathbb{SS}$	0.02 ± 0.02	0.03 ± 0.03	0.0014 ± 0.0014	0.0007 ± 0.0004
α	0.04 ± 0.03	0.11 ± 0.07	0.19 ± 0.02	0.096 ± 0.014
β	0.04 ± 0.03	0.27 ± 0.09	0.17 ± 0.02	0.17 ± 0.02
τ	0.12 ± 0.05	0.23 ± 0.09	0.106 ± 0.014	0.006 ± 0.004

Slope

	Q_{20}	Q_{60}	V_{60}	CATH
$\langle z \rangle$	0.17 ± 0.03 (0.000)	0.27 ± 0.05 (0.000)	-0.373 ± 0.017 (0.000)	0.425 ± 0.011 (0.000)
CO	1.19 ± 0.16 (0.000)	3.8 ± 0.3 (0.000)	2.7 ± 0.2 (0.000)	1.48 ± 0.13 (0.000)
$\mathbb{SS}$	-0.27 ± 0.09 (0.003)	0.4 ± 0.6 (0.080)	-0.18 ± 0.07 (0.013)	0.05 ± 0.09 (0.530)
α	-0.41 ± 0.11 (0.000)	-1.2 ± 0.2 (0.000)	-1.60 ± 0.04 (0.000)	-1.12 ± 0.04 (0.000)
β	0.30 ± 0.07 (0.000)	1.46 ± 0.16 (0.000)	1.22 ± 0.03 (0.000)	2.10 ± 0.06 (0.000)
τ	-0.95 ± 0.12 (0.000)	-2.8 ± 0.3 (0.000)	-3.64 ± 0.13 (0.000)	1.2 ± 0.2 (0.000)
